# Antibiotic-Mediated Modulation of the Gut Microbiome Identifies Taurine as a Modulator of Adipocyte Function Through TGR5 Signaling

**DOI:** 10.3390/ijms27020917

**Published:** 2026-01-16

**Authors:** Elisabeth Jäger, Viktoriya Peeva, Thorsten Gnad, Sven-Bastiaan Haange, Ulrike Rolle-Kampczyk, Claudia Stäubert, Petra Krumbholz, John T. Heiker, Claudia Gebhardt, Ute Krügel, Paromita Sen, Monika Harazin, Viktoria Stab, Julia Münzker, Nazha Hamdani, Alexander Pfeifer, Martin von Bergen, Andreas Till, Wiebke K. Fenske

**Affiliations:** 1 Division of Endocrinology, Nephrology, Rheumatology, Medical Department III, University Hospital of Leipzig, 04103 Leipzig, Germany; 2 Division of Endocrinology, Diabetes and Metabolism, Department of Internal Medicine I, University Medical Center Bonn, 53127 Bonn, Germany; vpeeva@uni-bonn.de (V.P.); paromita.sen.sg@gmail.com (P.S.); andreas.till@bfarm.de (A.T.); 3 Institute of Pharmacology and Toxicology, University Hospital, University of Bonn, 53127 Bonn, Germany; thorsten.gnad@bfarm.de (T.G.); alexander.pfeifer@uni-bonn.de (A.P.); 4 Federal Institute for Drugs and Medical Devices (BfArM), 53175 Bonn, Germany; 5 Department of Molecular Toxicology, Helmholtz Center for Environmental Research—UFZ, 04318 Leipzig, Germany; sven.haange@ufz.de (S.-B.H.); ulrike.rolle-kampczyk@ufz.de (U.R.-K.); martin.vonbergen@ufz.de (M.v.B.); 6 Rudolf Schönheimer Institute of Biochemistry, Faculty of Medicine, Leipzig University, 04103 Leipzig, Germany; claudia.staeubert@medizin.uni-leipzig.de (C.S.); petra.krumbholz@medizin.uni-leipzig.de (P.K.); 7 Helmholtz Institute for Metabolic, Obesity and Vascular Research (HI-MAG) of the Helmholtz Zentrum München at the University of Leipzig and University Hospital Leipzig, 04103 Leipzig, Germany; john.heiker@helmholtz-munich.de (J.T.H.); claudia.gebhardt@helmholtz-munich.de (C.G.); 8 Rudolf Boehm Institute of Pharmacology and Toxicology, Faculty of Medicine, Leipzig University, 04107 Leipzig, Germany; ute.kruegel@medizin.uni-leipzig.de; 9 Department of Medicine, Endocrinology and Diabetes, Bergmannsheil University Hospitals Bochum, Ruhr University Bochum, 44801 Bochum, Germany; monika.harazin@bergmannsheil.de (M.H.); viktoria.stab@bergmannsheil.de (V.S.); 10 Department of Cellular and Translational Physiology, Institute of Physiology, Molecular and Experimental Cardiology, Medical Faculty, Ruhr University Bochum, 44801 Bochum, Germany; nazha.hamdani@rub.de

**Keywords:** obesity, microbiome, taurine, TGR5, lipolysis, thermogenesis

## Abstract

Gut microbiota has emerged as a modulator of host metabolism and energy balance. However, the precise microbial metabolites mediating thermogenic activation in obesity remain largely undefined. We investigated the effect of antibiotic treatment under a high-fat diet on metabolites and its contribution to lipolysis and thermogenesis. Antibiotic treatment in high-fat diet-fed rats reduced adiposity and enhanced adaptive thermogenesis. Metabolomics revealed elevated taurine levels in the cecum content and plasma of antibiotic-treated animals, correlating with increased expressions of UCP1 and TGR5 in brown adipose tissue. Taurine enhanced lipolysis and oxygen consumption in mouse adipose tissue and human adipocytes. Thereby, taurine modulated lipolysis dependent on TGR5 signaling in adipose tissue. Human data confirmed that taurine promotes browning of white adipocytes and that acute cold exposure leads to a marked drop in circulating taurine, suggesting its rapid recruitment into thermogenic tissues. Besides its synthesis in the liver and dietary uptake, taurine can be a microbiota-derived metabolite that activates adipose thermogenesis and lipolysis through TGR5 and possibly taurine transporter-dependent mechanisms. These findings uncover a gut–adipose axis with therapeutic potential for metabolic disease.

## 1. Introduction

Obesity and its related comorbidities represent one of the major global health burdens of our time. This multifactorial condition arises from a complex interplay between genetic, psychosocial, and environmental factors, including dietary composition and the gut microbiome. While incretin-based pharmacotherapies now allow for substantial drug-induced weight loss, additional strategies are needed to enhance peripheral energy expenditure for long-term cardiometabolic risk reduction. In our study, we identified the non-proteinogenic amino acid taurine as a metabolically highly active metabolite that increases lipolysis and thermogenesis. Based on our data, we hypothesize that taurine availability in the body not only depends on de novo synthesis and dietary sources but also on microbiome composition.

The gut microbiome, largely composed of anaerobic bacterial species, has been recognized as a key determinant of metabolic homeostasis [1,2,3]. Obese individuals typically exhibit reduced microbial diversity [1] and distinct microbial gene profiles compared to their lean counterparts [4]. These alterations, termed dysbiosis, have been causally linked to obesity and metabolic disease in both humans and animal models [5,6,7]. Interventions such as co-housing [8,9] and fecal microbiota transfer (FMT) [5,10] can modify obesity and metabolic phenotype in rodent models. Moreover, transplantation of fecal microbiota from obese to lean mice or humans, as well as FMT from human twin pairs stably discordant for obesity into germ-free recipients, transmits the donor’s adiposity and metabolic phenotype [6,11,12]. Interestingly, even the metabolic benefits of bariatric surgery have been partially attributed to microbiota shifts [10,13,14,15,16].

Depleting the gut microbiota by oral antibiotics emerged as a popular method to explore systemic microbial effects on physiology and disease in rodent models and to study the role of bacteria in supporting cell functionality and signaling pathways after development. Studies in mice have shown that the depletion of the microbiota by antibiotics [17,18] and germ-free regimens results in the transformation of white adipose tissue into functional beige/brown adipose tissue, leading to reduced adiposity and improved metabolic phenotype [19]. Moreover, a recent treatment study of mice with antibiotics suggested that the presence of a healthy gut microbiome is required for uncoupling protein 1 (UCP1)-dependent thermogenesis during cold exposure [20].

The molecular signals mediating microbiota-induced thermogenesis remain elusive. We hypothesized that specific microbial metabolites might impact adipose tissue remodeling under HFD and antibiotic treatment. In this study, we identify taurine—a non-proteinogenic amino acid—as a top candidate metabolite elevated in cecal and systemic compartments after antibiotic-mediated microbiota modulation. Taurine has recently been implicated as a driver of healthy aging and resistance to metabolic disease [21], yet its mechanisms of action in adipose tissue remain poorly characterized. Here, we demonstrate that taurine positively correlates with adipose expression of UCP1 and Takeda G protein-coupled receptor 5 (TGR5, *GPBAR1*)—a bile acid-sensing G protein-coupled receptor known to regulate thermogenesis [22,23]. Using both mouse and human adipocyte models, we show that taurine promotes lipolysis and oxygen consumption and induces browning of white adipocytes. Taurine-triggered lipolysis is thereby reduced in adipose tissue from TGR5-deficient mice, suggesting a novel signaling pathway for taurine. Notably, taurine levels in the serum of human subjects rapidly decline during cold exposure, suggesting its physiological role in thermogenic recruitment. Together, these findings uncover taurine as a key regulator of thermogenesis and lipid mobilization, with potential translational relevance for obesity treatment.

## 2. Results

### 2.1. Antibiotic Treatment During High-Fat Diet Modulates Thermogenesis in Rats

To study the functional effect of gut microbiota dysbiosis under high-fat feeding conditions on host energy and metabolic control, eight-week-old Wistar rats were fed a high-fat diet (HF) for 12 weeks. Since the microbiome of rats was reported to be more similar to the human microbiome, rats were chosen for antibiotic treatment in our study [24]. Feeding regimens were continued after randomization into an antibiotic (ABx) or non-antibiotic (non-ABx) treatment arm for 35 days, as described before [10,25,26] (for details, see Materials and Methods and Supplementary Figure S3). Standard chow-fed rats with or without antibiotic treatment were kept as the control group (Supplementary Figure S1). The effects on energy control and glucose homeostasis were analyzed at the end of the 12-week treatment period.

**Figure 1:**
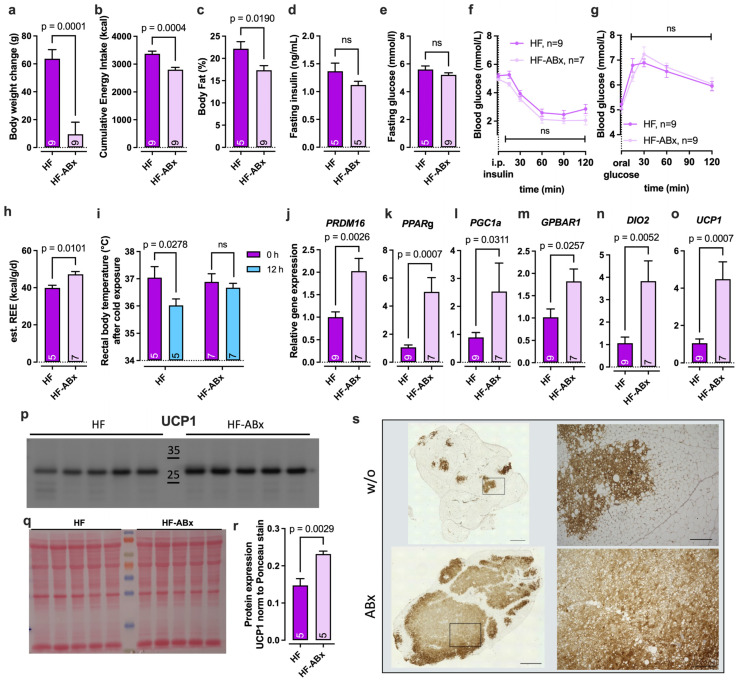
Antibiotic treatment alters the metabolic phenotype in high-fat diet-fed rats. (**a**–**s**) Male Wistar rats were fed a high-fat diet (HF), and drinking water was optionally supplemented with an antibiotic cocktail for 35 days (ABx) at the end of the 12-week experimental setup. Bars indicate mean + SEM; sample size as indicated within bars reflects the number of tissue samples from different animals. The Shapiro–Wilk test was used to determine normal distribution, and the unpaired t-test or Mann–Whitney U testing was used to calculate *p*-values, as indicated in graphs; ns indicates non-significant *p*-values. (**a**) Body weight change 35 days after indicated treatment; (**b**) cumulative energy intake over 35 days of treatment; (**c**) body fat measurement after 35 days of antibiotic treatment; (**d**) fasting insulin measurement; (**e**) fasting glucose measurement; (**f**) blood glucose measurement after intraperitoneal injection of insulin; (**g**) blood glucose measurement after oral glucose tolerance test; (**h**) estimated resting energy expenditure (eREE); (**i**) rectal body temperature after cold exposure for 12 h at 4 °C; (**j**–**o**) gene expression analysis via RT-qPCR of thermogenesis marker genes in brown adipose tissue (BAT) using beta-actin as a reference gene: relative gene expression shows comparisons of HF-ABx samples normalized to mean of HF samples; (**p**) detection of uncoupling protein 1 (UCP1) protein expression via Western blot in BAT: numbers and lines show molecular weight markers in kDa; (**q**) Ponceau stain for Western blot membrane in p; (**r**) quantification of UCP1 protein expression normalized to Ponceau stain via ImageJ; (**s**) UCP1 staining (IHC) of whole BAT sections (scale bar 1000 µm) and magnified sections (scale bar 200 µm) of black framed tiles in overview image.

Changes in body weight were measured after the 35-day antibiotic treatment period, and the data indicate that antibiotic treatment results in a significantly reduced body weight gain under high-fat diet feeding (Figure 1a), associated with a significantly lower cumulative energy intake compared to the HF group (Figure 1b). Accordingly, the body fat content of HF-ABx animals was also significantly reduced (Figure 1c). The analysis of interventional effects on glycemic control showed no significant difference in reduced fasting insulin (Figure 1d) and fasting glucose levels (Figure 1e) in the HF-ABx group compared to HF animals. Similarly, no significant difference in systemic insulin sensitivity (Figure 1f) nor in glucose tolerance (Figure 1g) was found.

Analyzing the effect of antibiotic treatment on energy metabolism, the estimated resting energy expenditure (eREE) was calculated as described before [10,27]. Antibiotic supplementation resulted in a significantly increased eREE in HF-ABx compared to HF animals (Figure 1h). Additionally, HF-ABx rats showed a significantly higher cold tolerance after cold exposure (4 °C) for 12 h and more efficient heat production during cold exposure (36.89 °C (t_0_) to 36.67 °C (t_12h_)) compared to HF controls (37.04 °C (t_0_) to 36.02 °C (t_12h_)) (Figure 1i). To characterize the transcriptional regulation of adaptive thermogenesis, the gene expression of markers of brown adipocyte differentiation (PRDM16 and PPARγ), adipocyte activation (PGC1α and PPARγ), and thermogenesis signaling (TGR5, DIO2, and UCP1) was analyzed via real-time quantitative PCR (RT-qPCR) in brown adipose tissue (BAT, Figure 1j-o). Gene expression in antibiotic-treated rats (HF-ABx) was normalized to the HF control group. All analyzed marker genes (*PRDM16*, *PPARg*, *PGC1a*, *GPBAR1*, *DIO2*, and *UCP1*) of brown adipocyte functions were found significantly upregulated after antibiotic treatment in HF animals compared to HF without antibiotics (Figure 1j–o). Protein expression of the master thermogenesis marker UCP1 in BAT was investigated by Western blotting (Figure 1p). Normalization to Ponceau stain (Figure 1q), as validated before [28], showed significantly increased protein levels secondary to antibiotic treatment in HF animals compared to the HF control (Figure 1r). Immunohistochemistry of UCP1 in BAT confirmed the results of Western blot analysis, showing a more prominent staining for UCP1 in HF-ABx compared to HF animals (Figure 1s).

Notably, the effects of antibiotic treatment on energy metabolism seen under HFD feeding conditions were less pronounced in animals fed with standard chow (Supplementary Figure S1). Body weight (Figure S1a), cumulative energy intake (Figure S1b), body fat (Figure S1c), eREE (Figure S1d), and cold tolerance (Figure S1e) were not affected by antibiotic treatment under standard chow (SC) feeding conditions. While the expression level of thermogenic gene markers in BAT was partially regulated by antibiotic treatment under SC conditions (Figure S1f–k), no effects on UCP1 protein expression (Figure S1l–o) were found using Western blotting (Figure S1l–n) or IHC staining for UCP1 (Figure S1o).

Besides increasing thermogenic capacity in BAT, antibiotic treatment in HFD-fed animals also affected adipocyte size distribution in subcutaneous white adipose tissue (sWAT, Figure S1p,q), and less pronounced in visceral white adipose tissue (vWAT, Figure S1r,s). HF animals featured a reduced number of small adipocytes (<5000 μm^2^) compared to SC animals (Figure S1q,s). Antibiotic treatment partially neutralized this effect on cell size seen under HF feeding conditions by promoting a higher percentage of smaller adipocytes and a reduced fraction of larger adipocytes in HF-ABx compared to HF animals (Figure S1q).

In summary, these data support existing data on the beneficial effects of antibiotic-induced microbiota modulation on adiposity and especially on BAT recruitment and thermogenic capacity under HF feeding conditions [19,29,30,31,32].

### 2.2. Gut Bacteria Analysis Reveals an Increased Abundance of the Genera Akkermansia and Bacteroides After Antibiotic Treatment of HF Diet-Fed Rats

**Figure 2:**
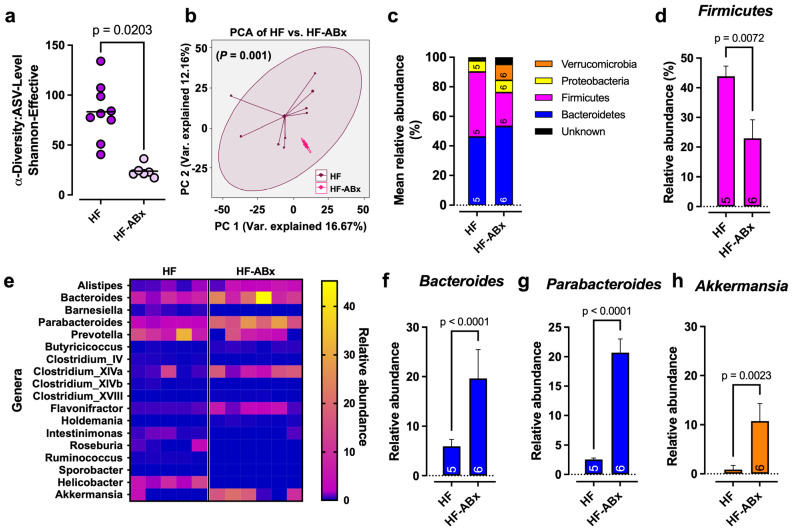
Antibiotic treatment of high-fat diet-fed rats shifts the gut microbiome to an enrichment of *Bacteroides*, *Parabacteriodes*, and *Akkermansia*. (**a**–**h**) Male Wistar rats were fed a high-fat diet (HF), and drinking water was optionally supplemented with an antibiotic cocktail (ABx) for 35 days. 16S rRNA analysis was performed on samples from the cecum content. Mann–Whitney U testing was used to calculate *p*-values, as indicated in graphs. (**a**) α diversity (variation in microbes per sample) is represented as the Shannon effective index (combination of richness and evenness); ASV—amplicon sequence variant. (**b**) α diversity (variation in microbial communities between samples) is shown for cecum samples from HF and HF-Abx; comparison by principal component analysis (PCA); *p*-values were calculated by PERMANOVA. (**c**) Distribution of phyla in cecum content is shown as mean relative abundance in stacked bars or as separate bar charts for *Firmicutes* in (**d**). Relative abundance refers to the total number of 16S rRNA gene reads in each sample. (**e**) Heatmap of relative abundance of genera in cecum content samples; light colors within the heat map represent high abundance of various genera. (**f**–**h**) Selected significantly (ANOVA) regulated genera from (**e**) as bar charts: *Bacteroides* (**f**), *Parabacteroides* (**g**), and *Akkermansia* (**h**).

To gain more specific insight into the antibiotic-mediated perturbation of the gut microbial environment, the cecal microbial composition from HF-fed animals with and without antibiotics was profiled. DNA isolated from cecum content was applied to 16S rRNA sequencing. The α-diversity decreased tremendously with antibiotic treatment (Figure 2a). Principal component analysis (PCA) confirmed the decreased variance in samples from ABx-rats, indicated by narrow clustering of samples, which was not observed in the control group (Figure 2b). The relative abundance analysis of bacterial phyla (Figure 2c) indicated a moderate enrichment of *Bacteroidetes* (Figure 2c) and a significant decline in *Firmicutes* (Figure 2d) in antibiotic-treated animals compared to the control group. Moreover, the phylum *Verrucomicrobia*, which was barely present in the HF group, appeared with approximately 10% relative abundance in the HF-ABx group (Figure 2c). To further delineate which bacterial genera correlate most with lower adiposity and increased adipose tissue thermogenesis after antibiotic treatment, we identified genera that were enriched specifically in cecum samples from HF-fed, antibiotic-treated rats (Figure 2e). Within the significantly enriched genera after antibiotics treatment were *Bacteroides* (Figure 2f) and *Parabacteroides* (Figure 2g) from the phylum *Bacteroidetes*, as well as *Akkermansia* from the phylum *Verrucomicrobia* (Figure 2h).

### 2.3. Metabolomics Analysis Uncovers Taurine and Taurine-Conjugated Bile Acids as Candidate Metabolites Promoting Adipocyte Thermogenesis Secondary to Microbiota Modulation Under HF Feeding

**Figure 3:**
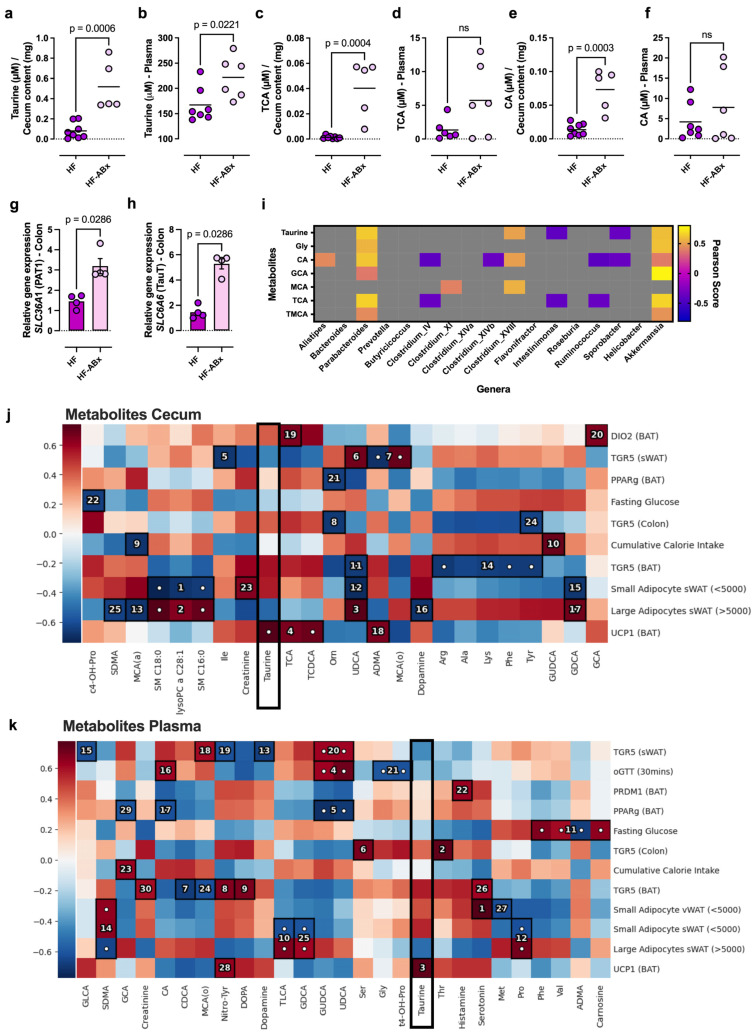
Metabolomics analysis reveals taurine as a potential metabolite relevant for the induction of adipose tissue thermogenesis. (**a**–**i**) Male Wistar rats were fed a high-fat diet (HF), and drinking water was optionally supplemented with an antibiotic cocktail (ABx). Metabolomics analysis via mass spectrometry was performed on samples from the cecum content and plasma. If not stated otherwise, the Mann–Whitney U test or unpaired *t*-test (if normally distributed data) was used to calculate significance levels, ns = non-significant. (**a**,**b**) Taurine concentration in cecum content ((**a**), *n* = 8/5) and plasma ((**b**), *n* = 7/6). (**c**,**d**) Taurocholic acid (TCA) concentration in cecum content ((**c**), *n = 8/5*) and plasma ((**d**), *n* = 6). (**e**,**f**) Cholic acid (CA) concentration in cecum content ((**e**), *n* = 8/5) and plasma ((**f**), *n* = 7/6). (**g**,**h**) Gene expression analysis of the taurine transporters *SLC36A1* (PAT1) ((**g**), *n* = 4) and *SLC6A6* (TauT) ((**h**), *n* = 4) in colon. (**i**) Correlation between metabolites and genera (16S analysis) in cecum content; the Pearson correlation score of significant correlations is shown: yellow tiles indicate positive correlation, blue tiles indicate negative correlations, and grey tiles indicate non-significant results. (**j**,**k**) Spearman’s rank correlation of phenotypic features and metabolites in cecum content (**j**) and plasma (**k**) (for lipid associations, see Figure S2a); top 30 correlations are indicated with numbers as rankings, white dots are significant correlations, red tiles are positive correlations, blue tiles are negative correlations, tiles without white dots are non-significant trends, and frames indicate equal rank correlation.

As microbiota-derived metabolites play a critical role in host–microbiota interactions related to energy balance and systemic metabolic control [33,34], we next analyzed metabolites in cecum content and plasma using mass spectrometry. Metabolomics data did not reveal a general pattern for the regulation of one of the analyzed metabolite groups (amino acids, biogenic amines, fatty acids, bile acids, acylcarnitine, sphingolipids, and glycerophospholipids). However, we observed antibiotic-dependent variations in abundance, specifically in some of the detected bile acids, including taurocholic acid (TCA) and cholic acid (CA), as well as taurine (Figure 3a–f). A striking enrichment was found for taurine in cecum content in the HF-ABx group compared to the HF group (Figure 3a), which was also detectable at significantly increased levels in the plasma of HF-ABx rats compared to HF controls (Figure 3b). The taurine-conjugated bile acid TCA was similarly increased in cecum content (Figure 3c) but less pronounced in the plasma (Figure 3d) of HF-ABx rats compared to HF control rats. Cholic acid, the unconjugated form of TCA, was also significantly increased in HF-ABx in cecum content (Figure 3e) but not in plasma (Figure 3f).

Since taurine levels were increased in both cecum content and plasma, we questioned whether the gene expression of taurine transporters is increased in the colon. Both the taurine transporter (TauT, *SLC6A6*, Figure 3g) and proton-coupled amino acid transporter 1 (PAT1, *SLC36A1*, Figure 3h) are known to be involved in intestinal taurine uptake. RT-qPCR analysis revealed a significant upregulation of both taurine transporters under HF-ABx conditions (Figure 3g,h), pointing towards a potential increased need for taurine recycling through the colon epithelium.

To analyze which of the bacterial genera listed in Figure 2e positively correlated with cecal taurine, bile acids, and related metabolites linked to taurine metabolism, a Pearson correlation analysis was performed. Significant correlation scores are shown in Figure 3g as colored tiles. Taurine positively correlated with the bacterial genera *Parabacteroides*, *Clostridium XVIII*, and *Akkermansia* (Figure 3i). Both *Akkermansia* and *Parabacteroides* were enriched after antibiotic treatment in the HF-ABx group (Figure 2g,h). Besides taurine, *Akkermansia* and *Parabacteroide* abundances also positively correlated with glycine, as well as with different conjugated (TCA and GCA) and unconjugated (CA) bile acid species (Figure 3i).

The joint results from the metabolite and correlation analysis led us to hypothesize that taurine and the taurine-conjugated bile acid TCA might be key players in the protection from HF diet-induced metabolic disturbances, as well as in the activation of adipose tissue thermogenesis under microbial control.

To test this hypothesis, we analyzed the association of measured metabolites in cecum content and plasma with metabolic and thermogenic parameters for all study groups using hierarchical association testing with the hierarchical all-against-all association testing (HAllA) method [35]. As expected, most plasma lipids were positively associated with large adipocyte size (Figure S2a). Importantly, HAllA revealed taurine in cecum content (Figure 3j) and plasma (Figure 3k) as a candidate (ranked fourth and third, respectively, by Spearman’s rank correlation) positively correlated with beneficial metabolic characteristics. These characteristics include the expression level of the thermogenic genes *UCP1* and *GPBAR1* in BAT, as well as small adipocyte sizes (<5000 μm^2^) in vWAT and sWAT. Taurine also showed a negative correlation with large adipocyte size in sWAT (>5000 μm^2^) and fasting glucose (Figure 3j). These associations suggest a potential role of taurine in lipolysis and thermogenesis. Although cecum TCA was also positively correlated with *UCP1* and *GPBAR1* gene expression in BAT, in plasma, this species missed a significant correlation with any metabolic feature, strengthening the case for taurine as a putative systemically relevant metabolic regulator.

Taurine, mainly synthesized in the liver through oxidation of cysteine by the enzyme cysteine dioxygenase (CDO), is one of the most abundant sulfur-containing amino acids found in organisms across eukaryotic phyla, with the highest concentrations in the heart (40 mM in rat heart), brain, retina, and muscles. Adipocytes were also shown to synthesize taurine by a CDO-dependent mechanism [36]. Plasma concentrations are within the micromolar range [37,38], with reported taurine plasma levels in rats and mice ranging from 0.25 mM to 1 mM [39]. A schematic overview of taurine metabolism is shown in Figure S2b. Hepatic conjugation of taurine to bile acids (e.g., TCA) by the bile acid-CoA:amino acid N-acyltransferase (BAAT) facilitates intestinal absorption of water-insoluble nutrients. Deconjugation and thereby liberation of taurine takes place in the gut via bile salt hydrolases (BSH) expressed by gut microbiota [40]. External sources for dietary taurine are mainly dairy, meat, and seafood, with taurine transporters facilitating its cellular uptake.

To further investigate whether antibiotic treatment affects the de novo biosynthesis or transport of taurine in the liver, we analyzed the gene expression levels of enzymes involved in taurine metabolism and its transport. RT-qPCR analyses of expression levels of *CDO*, *BACS*, *BAAT*, or *SLC6A6* in the liver were not affected by antibiotics (Figure S2c). Therefore, the increased serum taurine level might potentially derive from a higher recycling rate of taurine by an increased microbiota-mediated deconjugation of taurine-conjugated bile acids in the cecum rather than de novo synthesis in the liver.

These data point towards taurine as a metabolically active compound under antibiotic and presumably microbial regulation, with some bacteria, such as from the genus *Bacteroides* and *Parabacteroides* [41], bearing the capability to deconjugate taurine-conjugated bile acids, which results in the release of free taurine available for its recycling. Since taurine was associated with unique beneficial traits in our study, we focused our further analyses on the mechanism of taurine signaling and its direct effects on adipocyte function.

### 2.4. Taurine Modulates TGR5 Signaling In Vitro

In recent years, taurine has attracted significant attention for its beneficial metabolic and anti-obesity effects in various animal models [42,43], which were hypothesized to not only essentially result from lower caloric intake [44,45] but also from alterations in energy metabolism [36]. More recently, taurine deficiency has been proposed as a driver of aging in different species, and here, it is correlated with several disorders like obesity, metabolic syndrome, and mitochondrial dysfunction [21]. Its reversal was shown to increase health span in worms, rodents, and primates and to increase life span in worms and rodents [21].

Despite its numerous functional properties, the cellular and biochemical mechanisms mediating the actions of taurine remain incompletely understood. More specifically, the signaling mechanism of taurine mediating adipocyte lipolysis and the production of UCP1 remains completely unknown. Gene expression analyses of *SLC6A6* (TauT) in BAT only showed a trend in an increase in *SLC6A6* expression after ABx treatment (Figure S2d), indicating that *SLC6A6* expression in BAT is not underlying regulation induced by antibiotics treatment. Therefore, the positive correlation between systemic taurine abundance and the expression level of *GPBAR1* (TGR5) in BAT (Figure 3k) and its positive correlation with the characteristics of lipolysis and adipocyte thermogenesis attracted our attention.

TGR5 is well-known to be activated by a wide spectrum of taurine-conjugated and unconjugated bile acids, resulting in G_s_ protein signaling with subsequent cyclic AMP (cAMP) production [46]. The cAMP-dependent protein kinase A is fueled by cAMP, which activates downstream the transformation of tetraiodothyronine (T4) to triiodothyronine (T3) via DIO2, further promoting the upregulation of genes involved in energy consumption and adaptive thermogenesis (such as UCP1) in BAT and skeletal muscle. UCP1 uncouples respiration from the ATP synthase in the mitochondrial membrane, leading to increased energy expenditure and heat production [47].

**Figure 4:**
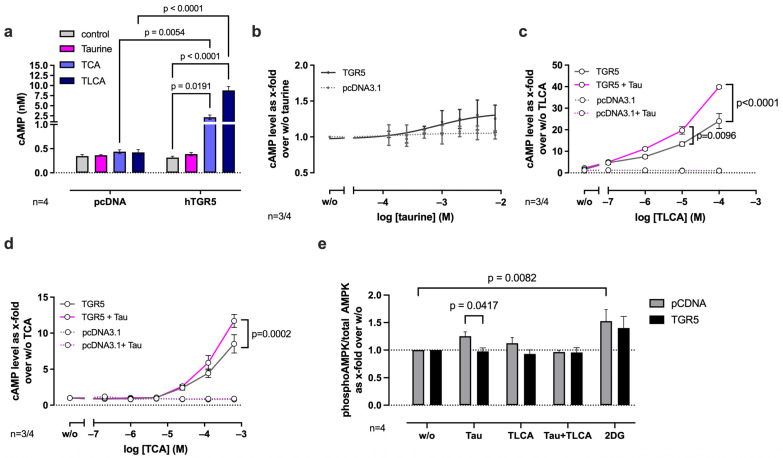
Taurine boosts TGR5 signaling towards cAMP production in vitro. HEK293-T cells were transfected with pTGR5 (human) or vector control (pcDNA3.1). (**a**) Measurement of cAMP production after stimulation with 2 mM taurine, 25 µM of taurolithocholic acid (TLCA), or 100 µM of taurocholic acid (TCA) for 30 min. (**b**) Measurement of cAMP production after stimulation with increasing concentrations of taurine, as indicated. (**c**) Measurement of cAMP production after stimulation with increasing concentrations of TLCA, as indicated, with or without addition of 0.5 mM taurine (Tau). (**d**) Measurement of cAMP production after stimulation with increasing concentrations of TCA, as indicated, with or without 0.5 mM taurine. (**e**) p—AMPK/AMPK detection after stimulation with 1 mM taurine and/or 100 µM of TLCA or 12.5 mM 2-deoxyglucose (2-DG) as a positive control. Data shown in (**b**–**e**) were normalized to baseline response. Symbols indicate mean ± SEM of 4 independent experiments. Two-way ANOVA plus Šídák test to correct for multiple comparisons was used for statistical testing.

To test whether taurine modulates TGR5 signaling in vitro, resulting in cAMP production, we stimulated human TGR5-transfected HEK293-T cells with taurine, as well as the TGR5 agonists taurolithocholic acid (TLCA) and TCA as positive controls. The stimulation of control-transfected cells (pcDNA3.1) with 25 µM of TLCA or 100 µM of TCA resulted in a slight (1.2×) but non-significant increase in cAMP production compared to unstimulated cells (Figure 4a). The stimulation of cells transiently expressing human TGR5 (hTGR5) with taurine, TCA, and TLCA resulted in a 1.25-fold, 2.1-fold, and 8.8-fold increase in cAMP production, respectively, thereby not reaching significance for taurine (Figure 4a). Interestingly, examining the dose–response curve of taurine normalized to background response (*w*/*o*) reveals a robust, concentration-dependent (125 μM–8 mM) effect of taurine on TGR5-transfected cells, which is not visible in control cells (Figure 4b). Although no significant effects were seen for taurine stimulation, we further asked whether taurine could modulate the response of TGR5 upon TCA or TLCA stimulation. We stimulated hTGR5-transfected cells with increasing concentrations of TLCA (100 nM–100 μM) in the presence or absence of 0.5 mM taurine (Figure 4c). At TLCA concentrations of 10 μM and 100 μM, the addition of taurine significantly increased cAMP levels by 1.5–1.6-fold (Figure 4c) without affecting control-transfected cells. A similar and significant increase in cAMP production (1.4-fold at 1 mM TCA) was seen when stimulating with TCA (40 nM–1 mM) in combination with 0.5 mM taurine (Figure 4d).

Given that previous studies reported AMP-activated protein kinase (AMPK) signaling upon taurine stimulation in myotubes [26] and white adipocytes [48], we questioned whether AMPK signaling is activated by taurine and mediated by TGR5. We used pcDNA/hTGR5-transfected HEK293-T cells and measured AMPK phosphorylation after stimulation with taurine and TLCA (Figure 4e). Interestingly, stimulation with taurine resulted in the slightly increased phosphorylation of AMPK in control-transfected cells, which was absent in hTGR5-transfected cells, indicating that taurine does not activate AMPK signaling through TGR5 (Figure 4e). Also, TLCA had no effects on AMPK phosphorylation. 2-deoxyglucose (2-DG), used as a positive control, stimulated AMPK signaling (Figure 4e). The results from these in vitro experiments reveal that taurine can modulate TCA/TLCA-activated TGR5 signaling towards cAMP production.

### 2.5. Taurine Modulates Lipolysis Through TGR5 Signaling in Mouse Adipose Tissue

**Figure 5:**
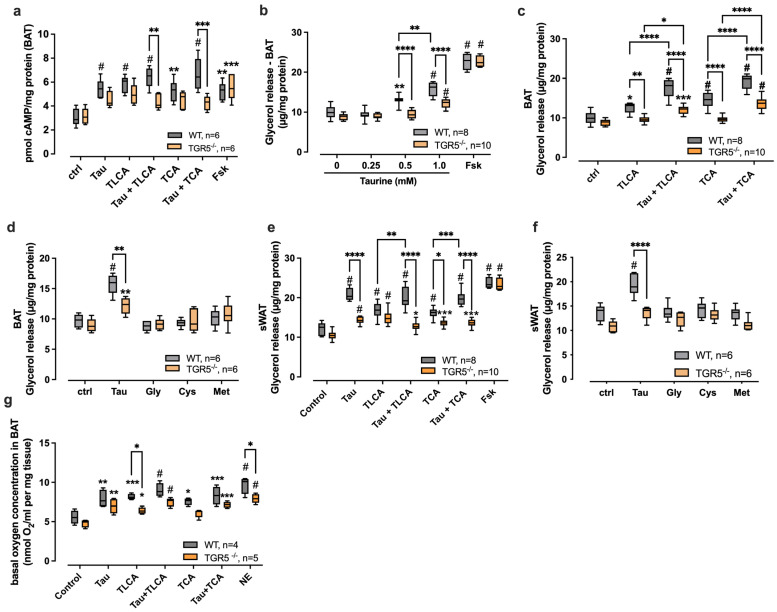
Taurine activates lipolysis in mouse adipose tissue by modulating the signaling of TGR5. (**a**) cAMP measurement of mouse brown adipose tissue (BAT) sample lysates from wild-type (WT) or TGR5-deficient mice (TGR5^−/−^) after stimulation with 1 mM taurine ± 100 μM taurocholic acid (TCA) or 25 μM of taurolithocholic acid (TLCA). In total, 1 μM of forskolin (Fsk) was used as a positive control. (**b**–**d**) BAT of TGR5^−/−^ mice and control mice (WT) was stimulated with increasing concentrations of taurine as indicated (**b**): 1 mM taurine, tauro-Lithocholic acid (TLCA, 25 µM) or/and taurocholic acid (TCA, 100 µM) (**c**) and with 1 mM taurine (Tau), glycine (Gly), cysteine (Cys), or methionine (Met) (**d**). Forskolin (Fsk, 1 µM) was used as a positive control. Glycerol release was measured and normalized to tissue weight; (**e**,**f**)—sWAT of TGR5^−/−^ mice and control mice was stimulated with 1 mM taurine, Tauro-Lithocholic acid (TLCA, 25 µM), or/and taurocholic acid (TCA, 100 µM) (**e**) and with 1 mM taurine (Tau), glycine (Gly), cysteine (Cys), or methionine (Met). (**f**) Forskolin (Fsk, 1 µM) was used as a positive control. Glycerol release was measured and normalized to tissue weight; (**g**) Measurement of basal oxygen consumption (oxygraph) of BAT of WT and TGR5-deficient mice (TGR5^−/−^) after stimulation with 1 mM taurine (Tau) or/and Tauro-Lithocholic acid (TLCA, 25 µM) or/and taurocholic acid (TCA, 100 µM). Norepinephrine (NE) was used as a positive control. (**a**–**g**) Box and whisker plots show 25th to 75th percentiles as box and whiskers by Tukey’s method. Sample size as indicated within graphs represent number of different tissue samples analyzed. Two-way ANOVA with correction for multiple comparisons (Sidak method) was used to calculate significance levels. If statistical comparison is not indicated by a line, the comparison is directed to the corresponding control stimulation (WT or TGR5^−/−^), only relevant comparisons are shown; * *p* < 0.05, ** *p* < 0.01, *** *p* < 0.001, and ****/# *p* < 0.0001.

To investigate whether taurine influences TGR5 signaling in metabolically active adipose tissue, we isolated adipose tissue from wild type (WT) and TGR5-deficient (TGR5^−/−^) mice and examined the response to taurine and selected bile acids. Initially, we measured cAMP in adipose tissue lysates after stimulation with taurine, TLCA, TCA, and their combination with taurine. The stimulation of isolated WT BAT with taurine resulted in a significant increase in intracellular cAMP levels (1.9 ± 0.4-fold over control), whereas the stimulation of BAT from TGR5^−/−^ mice with taurine did not result in a significant increase in cAMP production compared to the control (1.5 ± 0.2-fold over control) (Figure 5a). As seen for taurine, cAMP levels were significantly increased after the TLCA (2.0 ± 0.6-fold over control) and TCA (1.8 ± 0.5-fold over control) stimulation of WT BAT, without reaching significance in TGR5^−/−^ BAT (TLCA: 1.7 ± 0.5, TCA: 1.5 ± 0.5-fold over control). The combination of taurine with TLCA or TCA resulted in even more pronounced cAMP responses (TLCA + taurine: 2.2 ± 0.5, TCA + taurine: 2.3 ± 0.9-fold over control), which were significantly reduced in TGR5^−/−^ BAT samples (TLCA + taurine: 1.4 ± 0.4, TCA + taurine: 1.4 ± 0.3-fold over control). Interestingly, cAMP levels in TGR5^−/−^ BAT did not stay at baseline levels after stimulation with various stimuli, potentially indicating an alternative cAMP signaling pathway (Figure 5a). Forskolin (Fsk) directly activates adenylyl cyclase and thereby cAMP production, which was used as a TGR5-independent positive control. Fsk stimulation resulted in increased cAMP production with no difference between BAT from WT and TGR5^−/−^ mice (1.8-fold over control) (Figure 5a).

As modulation of TGR5 signaling by bile acids has been shown to induce UCP1-dependent lipolysis and thermogenesis in adipose tissue, we next tested whether taurine also induces lipolysis by activating TGR5 signaling and measured glycerol release from BAT after stimulation with increasing concentrations of taurine (Figure 5b). Stimulation with 250 μM taurine as the lowest tested concentration did not result in increased glycerol release, while 0.5 mM and 1 mM taurine led to a 1.3–1.6-fold concentration-dependent increase in glycerol release from WT BAT explants compared to unstimulated control (Figure 5b). Glycerol release at these taurine concentrations was significantly lower in TGR5^−/−^ BAT compared to WT, although still reaching a 1.4-fold increase in TGR5^−/−^ BAT compared to control at the highest tested taurine concentration (Figure 5b). The stimulation of lipolysis with forskolin resulted in a 2.2–2.6-fold increase in glycerol release, without a difference between WT and TGR5^−/−^ BAT (Figure 5b).

Our in vitro and ex vivo results reveal that taurine positively modulates TLCA- and TCA-triggered cAMP production, which we aimed to further study for the downstream activation of lipolysis. The stimulation of BAT explants with TLCA or TCA resulted in a 1.3- (TLCA) or 1.5-fold (TCA) mean increase in glycerol release in WT BAT, which was not seen in TGR5^−/−^ BAT (Figure 5c). The addition of taurine to TLCA or TCA resulted in a significant 1.8-fold (TLCA) or 1.9-fold (TCA) mean increase in glycerol release from WT BAT and a 1.4-fold (TLCA) or 1.5-fold (TCA) increase in TGR5^−/−^ BAT compared to the control and was significantly increased compared to stimulation with bile acid alone (Figure 5c). Lack of TGR5 in BAT resulted in a significantly decreased glycerol release compared to WT BAT when stimulated with taurine + TLCA/TCA (Figure 5c). In contrast to TCA/TLCA stimulation, which returns to baseline levels in TGR5^−/−^ BAT, additional taurine stimulation maintains the level of glycerol release above baseline, as observed with taurine alone (Figure 5b), suggesting an additional signaling mechanism independent of TGR5 for taurine.

To test the specificity of taurine on TGR5-mediated lipolysis in BAT, we included glycine (Gly) as the other amino acid conjugated to bile acids, as well as other sulfur-containing amino acids (cysteine (Cys) and methionine (Met)), in our study. While taurine significantly and partially TGR5-dependently increased glycerol release from BAT, none of the other tested amino acids could increase glycerol release in BAT (Figure 5d).

To study whether taurine induces lipolysis in white adipose tissue, we stimulated sWAT explants from WT and TGR5^−/−^ mice with taurine, as well as its combination with the bile acids TCA and TLCA. Similarly to BAT, taurine induced a 1.8 ± 0.4-fold increase of glycerol release compared to the control, which was significantly reduced in TGR5^−/−^ sWAT (Figure 5e). TLCA and TCA also significantly increased glycerol release, but surprisingly to a lesser extent (1.4-fold over control) compared to taurine (Figure 5e). Both TLCA- and TCA-induced glycerol release were only mildly affected by the absence of TGR5, with no significant decrease observed between WT and TGR5^−/−^ for TLCA (Figure 5e). The combination of TLCA and TCA with taurine resulted in a significant increase in glycerol release (1.7-fold over control), reaching similar levels as taurine alone, thereby not indicating any additional effect of TLCA and TCA in sWAT (Figure 5e). TGR5 deficiency in sWAT thereby also prevents the taurine-dependent increase in glycerol release when stimulated with the combination of taurine and TLCA/TCA (Figure 5e).

As seen in BAT, the stimulation of sWAT with forskolin led to a 2-fold increase in glycerol release, which is not affected by the absence of TGR5 (Figure 5e). Similarly to BAT, no other tested alternative amino acid (Gly, Cys, and Met) could induce glycerol release comparable to taurine in sWAT (Figure 5f).

Bile acid-induced activation of TGR5 signaling results in downstream activation of mitochondrial oxidative phosphorylation followed by increased energy expenditure in brown adipocytes [22,49]. To evaluate whether taurine induces oxidative phosphorylation in BAT through TGR5 signaling, we measured the basal oxygen consumption rate (OCR) in the presence of taurine, TCA, and TLCA (as the most potent TGR5-activating bile acid [46]). Basal respiration in WT BAT was significantly increased in the presence of taurine, TLCA, or TCA, but the combination of taurine and bile acids did not further increase basal respiration (Figure 5g). Whereas increases in basal respiration in the presence of TLCA/TCA depend on TGR5 signaling, taurine-triggered effects seemed to be independent of TGR5, as BAT from TGR5^−/−^ mice revealed no reduction in basal respiration compared to WT after taurine stimulation (Figure 5g). Norepinephrine (NE) stimulation was used as a positive control but was also reduced by the absence of TGR5 (Figure 5g), indicating a general respiratory deficiency in TGR5^−/−^ BAT.

In summary, our results from mouse adipose tissue explants show that taurine efficiently induces lipolysis in mouse BAT and sWAT and enhances TLCA and TCA-triggered lipolysis in BAT. Interestingly, taurine induces lipolysis to a similar level as seen with forskolin, thereby indicating almost maximal efficiency. Similar results were seen in basal respiration, where taurine enhances oxygen consumption in BAT to a similar degree compared to TLCA or TCA. Taurine-induced lipolysis thereby essentially depends on TGR5 signaling, whereas our data also indicate an alternative mechanism, as taurine still induces glycerol release to some extent in TGR5^−/−^ BAT and WAT. Interestingly, taurine-induced oxygen consumption appears not to be mediated through TGR5 signaling, indicating alternative signaling towards thermogenesis.

### 2.6. Taurine Enhances Lipolysis, Browning, and Respiratory Capacity in Human Adipocytes

**Figure 6:**
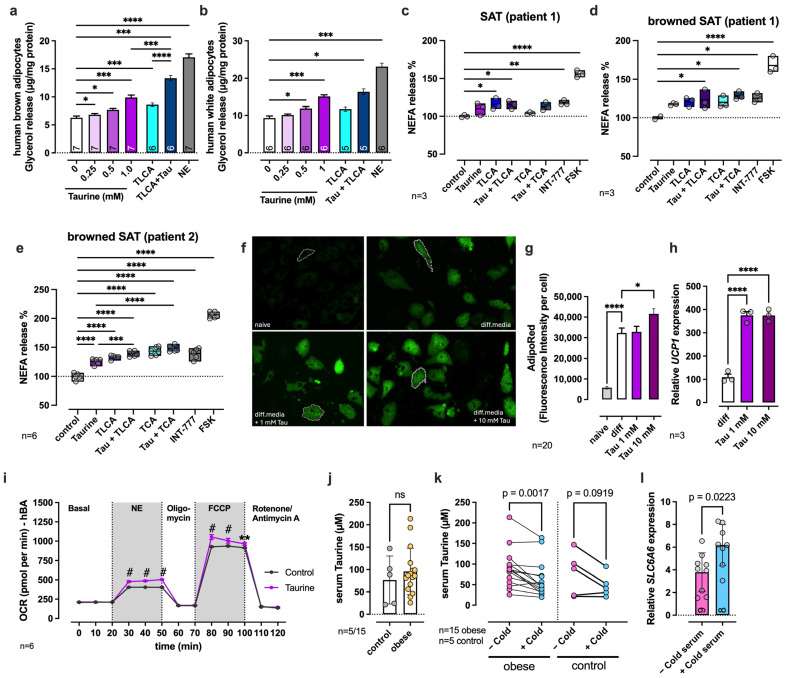
Taurine ameliorates lipolysis and mitochondrial efficiency in human adipocytes. (**a**,**b**) Human primary brown (**a**) or white (**b**) adipocytes were stimulated with increasing concentrations of taurine (0.25–1 mM), and glycerol release was measured and normalized to tissue weight. Norepinephrine (NE, 1 µM) was used as a positive control; (**c**–**e**) non-esterified fatty acid (NEFA) release in white (**c**) or browned (**d**,**e**) freshly isolated subcutaneous adipose tissue (SAT) from two obese patients after stimulation with 0.5 mM taurine, 100 µM TLCA, 100 µM TCA, 2 µM INT-777, and 1 µM Fsk; (**f**) AdipoRed stain after 28 days of differentiation of primary adipocytes with the indicated concentrations of taurine. (**g**) Quantification of AdipoRed stain fluorescence intensity as in (**f**) with ImageJ (IJ1.46r). (**h**) RT-qPCR analysis of *UCP1* gene expression after 28 days of adipocyte differentiation with 1 mM or 10 mM taurine. (**i**) Oxygen consumption rate was analyzed via Seahorse measurement in human primary brown adipocytes with or without 1 mM taurine and further addition of the indicated substances: NE (Norepinephrine, 1 µM), Oligomycin (2 µM), FCCP (1 µM), Rotenone (0.5 µM), and Antimycin A (0.5 µM). (**j**) Measurement of taurine in serum from lean (control) and obese individuals. (**k**) Analysis of taurine in serum before and after cold exposure of lean and obese individuals. (**l**) Stimulation of adipocytes with serum from (**k**) for 24 h and analysis of gene expression of *SLC6A6* (TauT). (**a**–**e**) Only relevant statistical comparisons are shown. One-way or two-way ANOVA, unpaired t-test (**j**,**l**), and paired *t*-test (**k**) were used for statistical testing: * *p* < 0.05, ** *p* < 0.01, *** *p* < 0.001, and #/**** *p* < 0.0001.

To confirm taurine as a metabolically active compound also in the human system, we stimulated human primary brown and white adipocytes with taurine and/or TLCA. As seen before in mouse adipose tissue, taurine increased glycerol release from primary brown (Figure 6a) and white (Figure 6b) human adipocytes in a dose-dependent manner, with a 1.6-fold increase at 1 mM taurine in brown (Figure 6a) and white adipocytes (Figure 6b). Stimulation with TLCA showed a similar increase in glycerol release in brown adipocytes as 0.5 mM taurine (1.3–1.4-fold over control), whereas the combination of TLCA and taurine significantly increased glycerol release compared to TLCA and taurine alone (2.1-fold (BA, Figure 6a)), confirming the additive effect seen in mouse BAT (Figure 5c). In contrast, no additive effect of taurine and TLCA on glycerol release was observed in white adipocytes (Figure 6b), a finding also seen in mouse sWAT (Figure 5e).

In accordance with these data, the stimulation of human adipocytes that were freshly isolated from sWAT from obese patients also revealed an increase in lipolysis (release of non-esterified fatty acids, NEFA) when stimulated with either taurine, TLCA, TCA, or the combination of taurine and bile acids (Figure 6c–e). Freshly isolated white adipocytes stimulated with 0.5 mM taurine resulted in a non-significant 10% increase in NEFA release, whereas a significant increase of 15%, 17%, and 56% was seen after stimulation with the positive controls TLCA, INT-777 (synthetic TGR5 agonist), and FSK, respectively (Figure 6c, patient 1). The efficiency of taurine-/bile acid-stimulated lipolysis increased when adipocytes were cultured in differentiation media for 7 days to trigger their differentiation into brown adipocytes (Figure 6d, e). Browning of adipocytes resulted in a 26% increase in NEFA release upon taurine stimulation (Figure 6e, patient 2) compared to the control. Stimulation with TLCA resulted in a 30% increase, and stimulation with TLCA + taurine resulted in a 39% increase in NEFA release compared to the control, which was even higher for TCA (43%) and TCA + taurine (48%) compared to the control (Figure 6e). The addition of the synthetic TGR5 agonist INT-777 had a slightly lower effect on lipolysis, with an increase of 40% compared to the control (Figure 6e). These data further confirm our results from mouse adipose tissue and highlight the physiological relevance of taurine in modulating lipolysis in human adipocytes.

To study whether taurine directly modulates browning during the differentiation of subcutaneous white adipocytes into brown adipocytes, taurine was added during the differentiation period of 32 days to the differentiation media. At the end of the differentiation period, intracellular lipid droplets were stained with AdipoRed (Figure 6f), and fluorescence intensity was quantified using ImageJ (Figure 6g). AdipoRed intensity significantly increased when adipocytes were differentiated in differentiation media (Figure 6g). While high (10 mM) taurine levels further increased AdipoRed intensity, the addition of 1 mM taurine did not have any further effect on AdipoRed intensity, indicating that the differentiation was already efficient with the differentiation media alone (Figure 6g). Interestingly, gene expression analysis showed that already lower levels of taurine (1 mM) increase UCP1 gene expression without any further increase with 10 mM taurine (Figure 6h), indicating a positive effect of taurine on the thermogenesis program.

To further analyze the effect of taurine on the thermogenic capacity of human brown adipocytes, we measured the oxygen consumption rate dependent on taurine availability. Treatment with 1 mM taurine increased norepinephrine (NE)-stimulated oxygen consumption and ATP synthase-uncoupled (Carbonyl cyanide 4-(trifluoromethoxy) phenylhydrazone, FCCP) oxygen consumption, indicating an increased thermogenic activity and expansion of maximal respiratory capacity in human brown adipocytes in the presence of taurine (Figure 6i).

As taurine abundance was postulated as a driver of aging and associated diseases, including obesity [21], we next investigated whether serum taurine levels diverge between obese and normal-weight human subjects, as has been reported before [50]. Interestingly, we did not see a significant difference in serum taurine concentrations between obese and normal-weight control subjects (Figure 6j). Instead, we found a significant drop in circulating taurine concentrations in both groups upon cold-induced thermogenesis (Figure 6k). For this experimental approach, human subjects were wrapped with cooling cuffs (circulating water temperature was 10 °C) for a period of 1 h, and peripheral blood was taken before and after the cooling process. A drastic decrease in serum taurine levels was seen in 19 out of 21 subjects, which are shown in Figure 6k, after cold exposure, independent of their body mass level (Figure 6k). These findings led us to hypothesize that circulating taurine might be rapidly recruited into thermogenic adipocytes (and potentially muscle) to facilitate thermogenesis during cold exposure, likely facilitated by the taurine transporter TauT. Cold-induced circulating factors might thereby activate the gene expression of the taurine transporter to facilitate its rapid uptake. To prove this idea, we stimulated differentiated adipocytes with serum taken before and after cold exposure for 24 h and measured the gene expression of *SLC6A6* (TauT) (Figure 6l). RT-qPCR results confirmed an upregulation of *SLC6A6* expression if adipocytes were stimulated with serum taken after cold exposure compared to stimulation with serum taken before cold exposure (Figure 6l).

In summary, our data identify taurine as a potential gut microbiota-derived activator of lipolysis and thermogenesis in BAT and WAT, which was demonstrated for mouse adipose tissue and human adipocytes. We hypothesize that taurine activates intracellular lipolysis at least partially by TGR5-mediated signaling and thermogenesis by a TGR5-independent mechanism. We observed a rapid decrease in circulating taurine during cold exposure of human subjects, indicating its need for thermogenesis in target organs. While TGR5 has been reported so far to be exclusively activated by bile acids, the combination of taurine with selected bile acids demonstrates additive effects on TGR5 signaling towards intracellular lipolysis, potentially indicating different receptor binding mechanisms rather than competitive binding to the same site.

## 3. Discussion

There is a growing consensus that the gut microbiome is a critical environmental regulator of human metabolic homeostasis and disease [33]. Moreover, changes in dietary regimens have been demonstrated to serve as central drivers of microbial composition and function, with the potential to affect the microbiome within days of initiation [51,52]. Remodeling gut dysbiosis by various means has gained growing interest as a promising therapeutic strategy in the management of obesity and metabolic diseases. The interaction between such strategies and dietary composition, as well as the underlying microbial signaling mechanisms regulating adipose tissue function, remains largely unclear.

In this study, we show that antibiotic-induced microbial depletion affects energy homeostasis. Specifically, under high-fat-diet (HF) conditions, antibiotics reduced weight gain and adiposity and enhanced thermogenesis via recruitment of brown adipose tissue (BAT). These effects were especially pronounced under high-fat-diet feeding and not as striking under SC feeding. Our findings are consistent with previous studies showing that antibiotic or germ-free conditions induce browning of white adipose tissue [19,29,30,32]. However, contrasting evidence has suggested that a healthy microbiota may be required for uncoupling protein 1 (UCP1)-dependent thermogenesis during cold exposure [19,29,30]. Our data support that microbiota control by antibiotic treatment can result in metabolic benefits, also depending on the dietary context.

We further identified an increased abundance of *Parabacteroides* and *Akkermansia* in the gut microbiota of HF-fed rats following antibiotic treatment. This is in agreement with earlier reports describing the enrichment of *Akkermansia* following antibiotic use [53,54], possibly due to its resistance to vancomycin, which was included in our antibiotic cocktail. Furthermore, increased mucus degradation following antibiotic exposure [55] might create a favorable niche for *Akkermansia* growth. *Akkermansia muciniphila* has previously been associated with beneficial metabolic effects, including improved glucose control and gut barrier function, as recently supported by a proof-of-concept clinical trial [56]. In our study, *Akkermansia* abundance positively correlated with increased levels of taurine in the cecum content of antibiotic-treated HF-fed animals, suggesting a not-yet-described potential link between this genus and microbial taurine metabolism.

Taurine, one of the most abundant amino acids in humans and other eukaryotes, has recently been linked with healthy aging in several species [21], and its supplementation in mice resulted in increased lifespan and was found to positively affect several hallmarks of aging, including cellular senescence, protection against telomerase deficiency, mitochondrial dysfunction, and attenuated inflammation. Interestingly, an association analysis of metabolites with clinical risk factors in humans showed that lower taurine levels were associated with adverse health, such as increased visceral adiposity, hypertension, inflammation, and type 2 diabetes [21]. Another recent preclinical study investigated the effect of taurine supplementation on exercise-linked metabolism in rats and reported that taurine supplementation improves endurance capacity with a delayed drop of blood glucose levels during exercise and increased lipolysis resulting in the release of free fatty acids as an energy source into the plasma [57]. Additional studies confirmed that taurine supplementation improves insulin sensitivity, glucose tolerance, and adaptive thermogenesis in mice [42,48,58] and also in humans [59], which is in accordance with our in vitro and ex vivo data and places taurine in the spotlight as a possible microbiota-derived driver of the improved metabolic phenotype described here.

Although various fundamental health benefits of taurine have been described within recent years, which essentially prepared the foundation for the ongoing first long-term, well-controlled taurine supplementation trials in humans, the cellular signaling mechanism(s) of how taurine affects cellular and organic health remain poorly understood. More specifically, its metabolic effects in adipocytes and targets of taurine signaling, especially in human adipocytes, are still missing.

We here demonstrate that taurine directly increases cAMP production, lipolysis, and oxygen consumption in mouse adipose tissue, as well as in primary human adipocytes. At the same time, our data unravel TGR5, a G protein-coupled receptor, as a novel mediator of taurine signaling towards lipolysis and demonstrate that taurine increases bile acid-activated receptor signaling through TGR5.

Previously, Guo and colleagues reported that the intraperitoneal treatment of HFD-fed mice with taurine activates AMPK in white adipocytes, resulting in increased PGC1α activation followed by increased oxygen consumption and induction of the thermogenic program [48]. Another in vitro study on white adipocytes from rats suggested that taurine stimulation positively modulates cAMP-dependent protein kinase A (PKA) signaling, leading to an increased β-adrenergic receptor (β-AR)-stimulated glycerol release [60], but in both studies, the receptor was not addressed. Therefore, we tested taurine-induced cAMP and AMPK signaling in TGR5-transfected HEK293 cells and excluded taurine-induced TGR5-dependent AMPK signaling (Figure 4e), but we could show that taurine modulates bile acid-induced, TGR5-dependent cAMP signaling in HEK293 cells (Figure 4).

TGR5 signaling has been well-characterized to be activated by a wide spectrum of conjugated and unconjugated bile acids [46]. Lithocholic acid (LCA) and its taurine-conjugated form (TLCA) have, among other bile acids, the highest potential to activate G protein stimulatory α subunit (Gα_s_) signaling and thereby intracellular cAMP production [46]. Watanabe et al. unraveled that bile acid-activated TGR5 signaling via the cAMP-type 2 iodothyronine deiodinase (DIO2) axis is involved in energy expenditure [22], which was further confirmed by TGR5 knockout mice, showing that the induction of browning of white adipose tissue and thermogenesis depends on this receptor [23]. Direct activation of TGR5 signaling with the synthetic agonist INT-777 further strengthened the described mechanism [23] and was shown to improve glycemic control in mice [49]. Here, we show that the addition of taurine to differentiating human brown adipocytes results in increased *UCP1* expression (Figure 6) by potentially feeding into the TGR5 signaling network and modulating thermogenesis in human adipocytes.

With these data, we link, for the first time, the already described beneficial effects of taurine in metabolism to TGR5 signaling. Our study showed that taurine itself can activate lipolysis in mouse BAT and sWAT, which was mainly TGR5-dependent (Figure 5). Importantly, experiments in human primary white and brown adipocytes, as well as adipocytes from obese patients, confirmed the physiological relevance of taurine signaling towards lipolysis in human adipocytes (Figure 6). The highest TGR5 receptor activation in mouse and human adipocytes was observed if both taurine and the tested bile acids were present, which may be explained by the two distinct cooperative agonist binding pockets described for TGR5 [61,62]. Bile acids can thereby bind to the orthosteric, large binding site of TGR5, whereas taurine may possibly bind to the second, allosteric binding site, which is important for agonist-driven cAMP accumulation [61,62]. However, the concept of ligand binding requires further investigation.

Interestingly, lipolysis induced by 1 mM taurine in sWAT almost achieved maximal levels, as seen with Fsk, and was higher than stimulation with the known most potent endogenous TGR5 agonists, TCA/TLCA. In accordance with this highly potent lipolytic activity, taurine was positively correlated with smaller adipocyte size in WAT in our association study (Figure 3j, k). Notably, however, taurine-activated cAMP production and lipolysis were partly still detectable in TGR5-deficient mouse adipose tissue in our setup. This finding suggests a complementary and TGR5-independent mechanism of taurine-mediated lipolysis, which may be mediated through AMPK signaling. AMPK signaling was shown before to be activated by taurine in adipocytes [48], as well as in myotubes [63], and was also found to be activated in our in vitro studies in HEK293 cells (Figure 4e).

In contrast to its lipolytic properties, oxygen consumption triggered by taurine was essentially independent of TGR5 in our experiments, indicating that an alternative mechanism is responsible for taurine-induced thermogenesis. Taurine transported into adipocytes via the taurine transporter directly affects mitochondrial respiration, as was shown before by taurine depletion in mice [64]. The gene expression of the taurine transporter TauT was partially increased in BAT in HF-ABx compared to HF animals (Figure S2d), which could promote an increased taurine supply for brown adipocytes, facilitating adaptive thermogenesis by a TGR5-independent mechanism. Interestingly, we found that circulating taurine levels immediately dropped during cold exposure in human subjects (Figure 6k). This drop in taurine levels is presumably caused by a rapid recruitment of taurine into thermogenic tissue facilitated by a cold-induced upregulation of TauT expression, as we could show by stimulating human adipocytes with serum taken before and after cold exposure (Figure 6l). Taurine might therefore potentially act on adipocytes through two distinct mechanisms to (1) induce lipolysis via TGR5 signaling and (2) enhance thermogenesis via TauT-mediated taurine influx. Thereby, the activation of TGR5 by taurine and bile acids may also lead to the trans-activation of TauT through PKA-mediated activating phosphorylation [65], but our data support an alternate TGR5-independent mechanism. The involvement of TauT in taurine-induced thermogenesis needs further validation, and experimental studies using validated inhibitors [66] will reveal the actual impact of TauT in taurine-activated thermogenesis.

These data confirm that antibiotic-induced microbiota control reduces weight gain and fat mass caused by HFD feeding while stimulating BAT recruitment and thermogenic capacity. Our study thereby provides an example for how a rational combination of dietary intervention with microbiome-targeting intervention may serve as a potential means of modulating physiological functions downstream of the microbiota. As such, we identified taurine as a fecal and circulating metabolite that increases during antibiotic treatment under high-fat nutrition, capable of activating and enhancing lipolysis and thermogenesis in both brown and white adipose tissue in mice, as well as in brown and white adipocytes in humans. We demonstrated that taurine induces lipolysis by modulating TGR5 signaling in an ex vivo setup, which needs to be confirmed by an in vivo taurine feeding study of WT and TGR5-deficient mice and optimally by gene knockdown in human adipocytes in vitro.

In summary, our findings reveal that the antibiotic-mediated remodeling of gut microbiota under high-fat dietary conditions induces an increase in taurine levels and improves the systemic metabolic phenotype. Taurine exerts dual effects: (1) promoting lipolysis through TGR5 modulation and (2) stimulating thermogenesis via a TGR5-independent mechanism that may involve TauT-mediated transport. These results suggest that taurine acts as a key effector metabolite in the gut–adipose axis and may serve as a promising therapeutic tool in the management of obesity and related cardiometabolic diseases.

## 4. Materials and Methods

### 4.1. Mouse and Rats

#### 4.1.1. Animals and Diets

Animal experiments were performed as described before [10], followed the international guidelines of animal care, and were approved by the local governmental authority for animal care (the state directorate of Saxony, Germany). Age-matched (8–10 weeks old) male Wistar rats (managed under the International Genetic Standardization/IGS program) were randomly allocated to experimental groups, based on their body weight as measured at the same circadian time throughout the experiments. Per group, 4–7 animals were assigned to 2–3 cohorts, depending on their feeding regimen, based on previous experience. Animals were individually housed on a 12h light/dark cycle in facilities with an ambient temperature of 21–23 °C and 40–60% humidity. Animals were fed standard chow (SC) (EF V1534-000, Ssniff Spezialdiäten GmbH, Soest, Germany) or a high-fat diet (HF) containing 58% of total energy as fat, 25.5% as carbohydrate, and 16.5% as protein (EF D12331, Ssniff Spezialdiäten GmbH, Soest, Germany) for seven weeks, followed by five weeks of the same diet with or without oral antibiotic treatment, as shown in the graphical overview in Supplementary Figure S3. Rats in the antibiotic group (ABx) received an antibiotic treatment (HF, SC) consisting of ampicillin (1 g/L; Ratiopharm, Ulm, Germany), vancomycin (0.5 g/L; Ratiopharm, Ulm, Germany), neomycin (1 g/L; Bela-pharm, Germany, Ulm, Germany), and metronidazole (1 g/L; CP-Pharma, Burgdorf, Germany), freshly provided every day via drinking water [10,25,26]. All antibiotics were given for a period of 5 weeks [26].

TGR5 knockout (KO) mice were kept on a C57BL/6 background and were kindly provided by Dr Verena Keitel (Heinrich-Heine-University, Düsseldorf, Germany) [67]. Heterozygous animals were used for breeding to obtain littermate TGR5 knockout and wildtype animals.

#### 4.1.2. In Vivo Metabolic Experiments

All in vivo experiments were performed as described before [10]. For the oral glucose tolerance test, the animals were fasted overnight, followed by an oral gavage of 20% dextrose (1 g/kg BW). Blood glucose was determined at 0, 15, 30, 60, and 120 min after glucose challenge (AccuChek Guide, Roche, Basel, Switzerland). Insulin tolerance tests were performed after overnight fasting conditions with Insulin administered intraperitoneally (0.5 U/kg BW). Calorie uptake was calculated via oxygen bomb calorimetry. The estimated total energy expenditure (TEE) was calculated by the energy balance method described by Chevalier et al. [27]. The following formula was used:

TEE (kcal/g) = energy intake (kcal/g) − 9.4 × Δfat mass + 1.8 × (kcal/g) × Δfat-free mass.

Differences in fat and lean mass were calculated from two DXA scan measurements during the first and last week of treatment. In total, 9.4 and 1.8 kcal/g are empirical values for the energy content of fat and lean mass, respectively.

#### 4.1.3. cAMP Measurement in BAT (As Described Before [68])

Adipocytes were stimulated as indicated for 15 min. Afterwards, cells were quickly washed with PBS and lysed with 0.1 M HCl. Subsequently, samples were analyzed using the Direct cAMP ELISA Kit (Enzo, Farmingdale, NY, USA, ADI-901-066), following the manufacturer’s instructions. Measurement of optical density was performed at 405 nm using a plate reader (Perkin Elmer, Shelton, CT, USA).

#### 4.1.4. Lipolysis Assay (As Described Before [68])

Differentiated adipocytes or adipose tissue explants were washed twice with lipolysis medium (Life Technologies, Carlsbad, CA, USA, DMEM21603) supplemented with 2% *w*/*v* fatty-acid-free BSA (Sigma-Aldrich, St. Louis, MO, USA, A7030), followed by incubation with lipolysis medium containing indicated substances at 37 °C and 5% CO_2_ for two (murine adipocytes and tissues) or four (human adipocytes) hours. Cell culture media were collected, 20 µL per sample was incubated for 5 min at 37 °C with 80 µL of free glycerol reagent (Sigma-Aldrich, F6428), and absorption was measured at 540 nm. Glycerol release was calculated with glycerol standard (Sigma-Aldrich, G7793) and normalized to protein content or wet tissue weight.

#### 4.1.5. Oxygen Consumption in Mouse Tissue Explants (As Described Before [68])

BAT and WAT were minced in 6-well plates and treated as indicated 15 min before oxygraphic measurements (Oxygraph 2K, Oroboros Instruments, Innsbruck, Austria). Samples were transferred to the oxygraph chamber containing 2 mL incubation medium (0.5 mM of EGTA, 3 mM MgCl_2_ 6H_2_O, 60 mM K-lactobionate, 10 mM KH_2_PO_4_, 20 mM HEPES, 110 mM sucrose, and 1 g l−1 bovine serum albumin (BSA), pH 7.1). Ex vivo endogenous respiration levels were recorded when reaching a steady state. Respiration rates were normalized to wet tissue weight.

#### 4.1.6. SDS Page/Western Blot

For WB, snap-frozen BAT samples were ground and prepared for SDS–PAGE using RIPA buffer supplemented with protease and phosphatase inhibitors (Roche, Basel, Switzerland). Then, 15 mg of total lysate was loaded on each lane and subsequently transferred to a nitrocellulose membrane. Blocking was performed with 5% BSA for 1 h at room temperature. UCP1 was detected using rabbit anti-UCP1 (Sigma-Aldrich, St. Louis, MO, USA, #ab23841) antibody incubated overnight at 4 °C. Horseradish peroxidase (HRP)-conjugated antibodies were used as secondary antibodies. Protein bands were detected with a Pierce TM ECL Western Blotting substrate (Thermo Fisher Scientific, Waltham, MA, USA) using the chemiluminescence detection method on G:BOX (Syngene, Cambridge, UK) and quantified with the corresponding GeneTools analysis software (v4.3.18, Syngene, Cambridge, UK).

#### 4.1.7. IHC Staining UCP1

Tissues were extracted, fixed in 4% paraformaldehyde (Sigma-Aldrich, St. Louis, MO, USA), paraffin-embedded, cut into 6 mm thick sections, and stained with H&E using standard techniques. Immunohistochemistry was carried out using rabbit anti-UCP1 (Sigma-Aldrich, St. Louis, MO, USA, #ab23841). All images were taken with Keyence (El Segundo, CA, USA) Fluorescence Microscope BZ-X800.

#### 4.1.8. Gene Expression Analysis via RT-qPCR

RT-qPCR was performed as described before [10]. The following primers were used on rodent cDNA:

rat_PRDM16-F: CCACACAGAAGAGCGTGAGTACAA;

rat_PRDM16-R: TGTGAACACCTTGACGCAGTTT;

rat_PPARG1-F: TGCCTTCGCTGATGCACTG;

rat_PPARG1-R: TGATCGCACTTTGGTATTCTTGG;

rat_PPARG2-F: ACAAGGACTACCCTTTACTGAAATTACC;

rat_PPARG2-R: GTCTTCATAGTGTGGAGCAGAAATGCTG;

rat_PGC1a-F: CACCAAACCCACAGAGAACAG;

rat_PGC1a-R: GGTGACTCTGGGGTCAGAG;

rat_TGR5-F: GTGCTTCGAGGAAGACCCAA;

rat_TGR5-R: AGTCCAAGTCAGTGCTGCAT;

rat_DIO2-F: GCACAGGAGACTGACTGAGG;

rat_DIO2-R: AATTTAACCTGTTTGTAGGCGTC;

rat_UCP1-F: CAACACTGTGGAAAGGGAC;

rat_UCP1-R: TGAGGTCATATGTCACCAGC;

rat_PAT1-F: CCACTGGGGAAGGCGCATC;

rat_PAT1-R: TGGGCGTTAGGATCACGGTC;

rat_TauT-F: CATCCGCTGTGGGCTTAG;

rat_TauT-R: CCATCTCCTCGTTTTGCTTG.

As a reference gene, beta-Actin was used:

rat_b-Actin-F: CATTGCTGACAGGATGCAGA;

rat_b-Actin-R: CTGATCCACATCTGCTGGAA.

#### 4.1.9. Microbiome Analysis (As Described Before [10])

Frozen cecum content samples were processed for bacterial DNA isolation using the QIAamp Stool Mini Kit (Qiagen, Hilden, Germany) according to the manufacturer’s instructions. Total bacterial content was analyzed exemplarily (n = 3 animals) by real-time PCR using bacterial 16S rDNA-specific primers (bacteria-16S-F: TCCTACGGGAGGCAGCAGT; bacteria-16S-R: GGACTACCAGGGTATCTAATCCTGTT). In short, DNA was isolated from weighted fecal samples, and RT-qPCR was performed in triplicate using fixed eluate volumes. Signal intensities were first transformed into expression levels by reversal of the log-transformation and then normalized to the used fecal mass by dividing by the correction factor (ratio of fecal masses). Normalized bacterial DNA content (mean of triplicate measurements) was displayed on a logarithmic scale.

For analysis of community composition, bacterial DNA samples were submitted to BGI (Hong Kong) for bacterial 16S DNA V3-V4 amplicon sequencing using the Illumina MiSeq PE300 platform. The following primers were used for library preparation: forward primer: 341F: ACTCCTACGGGAGGCAGCAG; reverse primer: 806R: GGACTACHVGGGTWTCTAAT.

Sequencing resulted in demultiplexed sequencing data. For each sample, two files in fastq format were provided: one for the forward reads and one for the reverse reads. Using the DADA2 R-package, the quality of reads was checked. Forward reads were trimmed at base pair position 280, and reverse reads were trimmed at base pair position 200. DADA2 was used to learn error rates, followed by filtering, denoising, merging of paired reads, and constructing amplicon sequence variants (ASVs). Taxonomy was assigned to ASVs using the DADA2 package in combination with the Ribosomal Database Project (RDP) database (http://rdp.cme.msu.edu/index.jsp, accessed 2019). The RDP data bases used where specifically formatted for DADA2 R-package and were the same versions found here https://zenodo.org/records/801828.

Normalization of ASV read counts, determination of alpha-diversity indices, and calculation of relative abundance for each taxonomic level were accomplished with Rhea. Significant differences in alpha-diversity indices and relative abundance of taxa between sample groups were determined by the Kruskal–Wallis Test with the Benjamini–Hochberg method for multiple comparisons where appropriate (independent tests > 20), followed by post hoc pairwise statistical analysis using the Dunn test. Beta-diversity between samples was analyzed by non-metric multidimensional scaling (NMDS) dissimilarity analysis, and global significant differences between sample groups were calculated by PERMANOVA using the Adonis function from the vegan R-package. The figures were constructed using the ggplot2 R-package.

#### 4.1.10. Metabolite Analysis: (As Described Before [10])

Mass Spectrometric Measurements: Bile acid measurements were carried out with the AbsoluteIDQ Bile Acid Kit (Biocrates Life Sciences GmbH, Innsbruck, Austria) according to the manufacturer’s instructions. The liquid chromatography–mass spectrometry (LC-MS/MS) analysis was carried out by MRM acquisition on a Waters Acquity UPLC System coupled to a QTRAP 5500 mass spectrometer (AB Sciex, Marlborough, MA, USA). Data processing was performed with the provided quantification method Kit (Biocrates Life Sciences AG).

Amino acids, amines, and selected lipid groups (lysophosphatidylcholines, phosphatidylcholines, and sphingolipids) were analyzed with the AbsoluteIDQ p180 Kit (Biocrates Life Sciences AG). Measurements were performed on a QTRAP mass spectrometer (MS) applying electrospray ionization (ESI) on a QTRAP 5500 mass spectrometer (AB Sciex). The MS was coupled to a UPLC (Waters Acquity, Waters Corporation, Milford, MA, USA). The metabolites were separated by a hyphenated reverse phase column (Agilent, Santa Clara, CA, USA, Zorbax Eclipse XDB C18, 3.0 × 100 mm, 3.5 µm) preceded by a precolumn (Security Guard, Phenomenex, C18, 4 × 3.0 mm; Phenomenex), applying gradient identification. Quantification was achieved by multi-reaction monitoring (MRM), standardized by applying spiked-in isotopically labelled standards in positive and negative modes. Data processing and metabolite quantification were performed with MetIDQ software vOxygen (Biocrates Life Sciences AG).

For short-chain fatty acid (SCFA) quantification, the method described by Han et al. [69] was used with some modifications. Plasma samples were mixed with acetonitrile to a final concentration of 50% acetonitrile and derivatized with 3-nitrophenylhydrazine. The mix was afterwards diluted 1:50 in 10% acetonitrile and injected into the LC-MS/MS system. Chromatographic separation of SCFAs was performed on an Acquity UPLC BEH C18 column (1.7 mm) (Waters) using H_2_O (0.01% FA) and acetonitrile (0.01% FA) as the mobile phases. For identification and quantification, a scheduled MRM method was used, with specific transitions for every SCFA.

For the quantification of cecal content samples, approximately 10 mg of each sample was used. Samples were processed with steel balls and extraction medium (acetonitrile–water; 1:1) in a ball mill to extract metabolites for downstream processing as described for plasma.

#### 4.1.11. Correlation Analysis

For correlation analysis between metabolites in the plasma or cecum and physiological parameters, a structured association discovery method between paired high dimensional datasets: Hierarchical all-against-all association (HAllA) testing was used (version 0.8.7) as described before [35], with Spearman’s rank correlation as the correlation metric, medoid as the clustering method, and q < 0.1 as the threshold for significance. Briefly, the HAllA algorithm follows the following steps: (i) optionally discretizing features into a uniform representation if necessary for the similarity measure; (ii) calculating the BH false discovery rate threshold; (iii) performing hierarchical clustering on each dataset separately to create two data hierarchies; (iv) pairing clusters with equivalent resolution from both hierarchies; (v) testing the paired clusters for statistically significant associations in block format and ensuring the block passes the false-negative tolerance (FNT) threshold; and (vi) gradually increasing the resolution by moving down through the hierarchies, selecting splits that result in the highest Gini score gain. The final feature groups are those that form the largest hypothesis blocks that meet the FNT threshold.

### 4.2. Human

#### 4.2.1. Isolation and Culture of Human Brown Adipocytes (As Described Before [70])

Primary human BA were isolated as described [71] and cultured in 60 mm culture dishes containing DMEM/F12, 10% FBS, 1% Penicillin/Streptomycin (all from Invitrogen, Carlsbad, CA, USA), and 1 nM acidic Fibroblast growth factor 1 (FGF-1 (ImmunoTools, Friesoythe, Germany). Cells were incubated at 37 °C with 5% CO_2_. Adipocytes were induced two days after full confluence with DMEM/F12 containing 1% Penicillin/Streptomycin, 0.1 mM dexamethasone (Sigma-Aldrich), 100 nM insulin, 200 nM rosiglitazone (Sigma-Aldrich), 540 mM isobutylmethylxanthine (Sigma-Aldrich), 2 nM T3 (Sigma-Aldrich), and 10 mg/mL transferrin (Sigma-Aldrich). After three days of differentiation, isobutylmethylxanthine was removed from the cell culture media. The cell cultures were left to differentiate for an additional nine days.

#### 4.2.2. Sea Horse Analysis of Human Brown Adipocytes (As Described Before [68])

The oxygen consumption rate in adipocytes was measured to evaluate oxidative phosphorylation using the Agilent Seahorse XFe24 Analyzers (Agilent Technologies, Santa Clara, CA, USA) following the manufacturer’s operating instructions. In brief, 2–4  ×  10^4^ cells were seeded in wells of a 24-well XF Cell Culture Microplate (Agilent Technologies, Santa Clara, CA, USA, 100777-004). Cells were grown in growth medium until they reached confluence, and then, they were differentiated into mature adipocytes. Assays were performed on mature adipocytes. The medium was exchanged to XF DMEM medium pH 7.4 (Agilent Technologies, 103575-100) with the addition of the following compounds: 25 mM glucose (G8270, Sigma-Aldrich, St. Louis, MO, USA), 2 mM glutamine (G9003, Sigma-Aldrich), and 2 mM sodium-pyruvate (P5280, Sigma-Aldrich). Cells were incubated in this medium for 1 h at 37 °C without CO_2_. Oxygen consumption rate measurements were performed with the addition of the indicated taurine and bile acids concentrations and by the sequential addition of 2 µM of oligomycin, 1 µM of FCCP, and 0.5 µM of rotenone/antimycin (Agilent Technologies, 103015-100). All the above experimental procedures were carried out at 37 °C. The basal and uncoupled respiration were determined, and the results are expressed in pmol/min and normalized to the cell number.

#### 4.2.3. Human Subcutaneous Adipose Tissue Collection, Isolation of Mature Adipocytes, and Cultivation as Membrane Aggregate Cultures

Subcutaneous adipose tissue (SAT) samples were collected during elective aesthetic and post-bariatric surgery at the Division of Plastic, Aesthetic, and Special Hand Surgery of University Hospital Leipzig. SAT was collected from the thighs during the thigh lift. All operations were performed under general anesthesia; the use of local anesthetics excluded patients from enrollment in this study. Patients with prior liposuction or cryolipolysis to the respective area were excluded from sample collection. Electrocautery was used to prepare the subcutaneous tissue for resection. Thermally damaged tissue and skin were removed using scissors or a scalpel, and fat samples were placed into sterile sample containers for immediate processing. Connective tissue was removed, and AT was processed with scissors to obtain a homogenous mixture that was digested with collagenase (500 u/g AT, Type II, Gibco, Waltham, MA, USA) for 45 min at 37 °C in an adipocyte isolation buffer (100 mM HEPES, 123 mM NaCl, 5 mM KCl, 1.3 mM CaCl_2_, 5 mM Glucose, 1% ZellShield^®^, 4% BSA). The digested AT was passed through a 300 μm syringe strainer (pluriSelect, Leipzig, Germany) and incubated at RT for approximately 5 min until a layer of mature adipocytes floating on top of the aqueous stromal vascular fraction became visible. Mature adipocytes were repeatedly washed in a Krebs–Henseleit Buffer (KHB; 25 mM HEPES, 10 mM CaCl_2_, 1% fatty acid-free BSA, pH 7.2) before cultivation under cell culture plate inserts (VWR, Radnor, PA, USA) [72]. In a 6-well format, up to 500 μL of adipocytes per well was cultured in 4 mL of DMEM/F-12 medium (supplemented with 10% FBS Superior and 1% ZellShield) at 5% CO_2_ and 37 °C. Adipocytes were cultured for 7 days without a change in medium. For adipocyte browning, cells were cultivated in browning medium (DMEM/F12 supplemented with superior 10% FBS, 1% Zellshield, 1 µM of rosiglitazone, 2 nM of triiodothyronine, and 20 nM of insulin) for 7 days. The medium was changed every 48 h.

#### 4.2.4. NEFA Measurement in Human Adipocytes

Human adipocytes were cultivated for 7 days with or without browning medium. On day 8, cells were harvested and washed with the Krebs–Ringer Bicarbonat buffer (KRBB; 120 mM NaCl, 25 mM NaHCO_3_, 5 mM KCl, 2 mM CaCl_2_, 1 mM MgCl_2_, 25 mM HEPES, 2 mM Glucose, 2% fatty acid-free BSA, pH 7.4). Then, 10 µL of adipocyte suspension was transferred into 90 µL of KRBB (with indicated stimulants) in a 96-well format and incubated for 4 h at 37 °C and 5% CO_2_. Subsequently, 10 µL of media samples without cells was collected and subjected to NEFA measurement using a commercially available assay (FUJIFILM Wako, Osaka, Japan).

#### 4.2.5. Taurine Measurement in Blood Before and After Cold Exposure

For cold exposure experiments, patients were placed in a resting position and wrapped with large surface cuffs connected to a water-circulating cooling system (Hilotherm clinic, Germany). The cuffs were cooled to 10 °C for a period of 1 h.

Free taurine levels in serum samples were measured using the Taurine Assay Kit from Cell Biolabs (San Diego, CA, USA, MET-5071) according to the manufacturer’s protocol. Before applying onto the test plate, the samples were deproteinated using centrifugal filter units Sartorius Vivaspin 20, 10,000 MWCO PES (REF VS2002). The centrifugation was performed for 30 min at 4000× *g*, 4 °C, and the obtained flow-through was then applied to the test plate. The assay was carried out using duplicates of undiluted samples. Studies involving human subjects were reviewed and approved by the local Ethics committee of the University Hospital of Bonn, Germany (proposal number 251/21). Informed consent was obtained from all subjects participating in the studies.

#### 4.2.6. Browning Assay Human Pre-Adipocytes

Human subcutaneous preadipocyte cells (#PT-5020, Lonza, Basel, Switzerland) were revived and expanded according to the protocols from Lonza with the PGM-2 BulletKit (Lonza, Basel, Switzerland, PT-8002). Their differentiation to brown adipocytes was carried out in a medium combined from the Lonza protocols and the recipe from Bartesaghi S. et al. [73]. One day before triggering the differentiation, the cells were seeded in 6-well dishes, 3 × 10^5^ cells per well. One 6-well dish was used per condition. Then, 90% confluent preadipocytes were treated with the following differentiation medium: DMEM/F12 (Gibco) with 3% fetal calf serum (Sigma) supplemented with one SingleQuot (Lonza, PT-9502 SingleQuots) per 200 mL medium: dexamethasone, 3-isobutyl-1-methyxanthine and insulin, and 5 nM triiodothyronine (Thermo Scientific). Then, 100 nM Rosiglitazone (R&D Systems, Minneapolis, MN, USA) was used to promote beige adipogenesis. The medium was exchanged every 7th day until fully differentiated (at day 32). Different components of interest, such as Taurine (1 mM, 10 mM, Sigma-Aldrich) and INT-777 (2 µM, Cayman Chemical Company, Ann Arbor, MI, USA), were added to the medium from day one of the differentiation until the end. Then, 1 of the 6 wells of the 6-well dishes was used for a differentiation checkup at day 15. Adipogenesis was confirmed by AdipoRed Assay Reagent (Lonza, Basel, Switzerland) on live cells. AdipoRed was also used on day 32 for microscopy, confirming the cells’ differentiation. Pictures were taken with Evos FLoid Invitrogen. For measuring the fluorescence of AdipoRed by ImageJ (version IJ1.46r), twenty cells per condition were selected using the drawing/selection tools. Area integrated, intensity, and mean grey value must be selected for the analysis. Three regions close to each cell were selected in a bubble as a background reference. The corrected total cell fluorescence was measured, and a mean value between all 20 cells was calculated and compared to the other conditions. Upon full differentiation, cells were collected as usual: 1×DPBS (Thermo Scientific) washing, trypsinization (0.25% Trypsin-EDTA (1x), Gibco), and addition of 2 mL growth medium per well. The cells were pulled together and centrifuged at 300× *g* for 5 min. The cell pellets were stored at −80 °C for RNA isolation. RNA was isolated with the RNeasy Plus Kit (Qiagen, Hilden, Germany). The samples were reverse transcribed with QuantiTect Reverse Transcription Kit (Qiagen, Hilden, Germany). qPCRs were performed on a LightCycler 96 System (Roche, Basel, Switzerland) and were analyzed with the provided LightCycler Software (v 1.1.0.1320). The following primers from Eurofins were used with the Blue S’Green qPCR 2x mix (Biozyme, Saint Joseph, MO, USA) at 60 °C, with 60 sec elongation time and 45 cycles:

TGR5-F: CCTGGCAAGCCTCATCATCA;

TGR5-R: CCAGCAGTAGGCTCAGGAAG;

TauT-F: AAGCCCAGAGGACAAGCTG;

TauT-R: TTTGGGAGCTCCTCAGAAGG;

B2M-F: CCACTGAAAAAGATGAGTATGCCT;

B2M-R: CCAATCCAAATGCGGCATCTTCA;

UCP1-F: CGGCTTTCTTCAAGGGGTTGG;

UCP1-R: GGCACAGTCCATAGTCTGCCT;

CIDEA-F: CCTCATCAGGCCCCTGACAT;

CIDEA-R: GAGGGCATCCAGAGTCTTGC;

PPARγ-F: AAAGGATGCGCTCTCGTTCA;

PPARγ-R: GGAATATGGTGATCGGGAACA;

CKMT1b-F: CAGAGGGCATCCTGTGAGCA;

CKMT1b-R: GCCGGTGCCAATCAGACTTTG.

#### 4.2.7. Transfection of Mammalian Cells

CHO-K1 cells: For transient transfection, Lipofectamine^TM^ 2000 (Thermo Fisher Scientific) was used. In total, 9 × 10^5^ cells were seeded into T25 cell culture flasks and transfected with a total amount of 3 μg of plasmid the following day. Cells were harvested and used for experiments 48 h after transfection. HEK293-T cells: For transient transfection, Lipofectamine^TM^ 2000 was used. Then, 1.4 × 10^6^ cells were seeded into T25 cell culture flasks and transfected with a total amount of 4 μg of plasmid the following day. Cells were harvested and used for experiments 48 h after transfection.

#### 4.2.8. Intracellular cAMP Measurement in HEK293 Cells

Intracellular cAMP in transfected HEK293 cells was measured as described in detail here [74] using the Perkin Elmer alpha screen assay following the manufacturer’s instructions.

#### 4.2.9. Multiplex Surefire Ultra Human Phospho-AMPKa1/2 (Thr172 and Total) Detection Assay

pAMPK/total AMPK content of HEK293-T cell extracts was determined by the Multiplex SureFire Ultra human phospho-AMPKa1/2 (Thr172 and total) detection assay according to the manufacturer’s protocol (revvity, Waltham, MA, USA). The kit measures both the phosphorylation (Thr172) and total levels of endogenous AMPKa1/2 in cellular lysates. The signal at 615 nm (Eu) corresponds to the phosphorylated AMPK level, and the signal at 545 nm (Tb) corresponds to the total AMPK levels. HEK293-T cells were split into 96-well plates 24 h after transfection and set on the serum-free medium overnight. Stimulation with agonists was performed 48 h after transfection in HBSS/HEPES (pH 7.4) for 60 min at 37 °C. Reactions were stopped by the aspiration of media, and cells were lysed in 50 μL of the supplied lysis buffer. From each well, 10 μL of lysate was transferred to a 384-well plate. Then, 5 µL of acceptor beads was added and incubated for 1 h at room temperature. Subsequently, 5 µL of donor beads was added according to the manufacturer’s protocol and incubated for 1 h at room temperature. The measurements were taken using the EnVision Microplate reader (revvity, Waltham, MA, USA).

#### 4.2.10. Statistics

All statistical analyses were performed using the software GraphPad Prism 10. Data are expressed as mean +/− SEM unless otherwise stated. Comparisons between two groups were performed using the Mann–Whitney U test or t-test if normal distribution was verified using Shapiro–Wilk-testing. ANOVA with corrections for multiple comparisons (Holm–Šídák method) was used for comparison between multiple groups where indicated. The numbers per group in the figure legends/figures refer to the number of subjects per group. Individual data points/animals were excluded manually if technical issues or abnormalities were detected during the procedures. Statistical significance is indicated by *p*-value: * *p* < 0.05, ** *p* < 0.01, *** *p* < 0.001, and ****/# *p* < 0.0001.

#### 4.2.11. Resource Availability

Resources are available upon request from the corresponding author, Wiebke Kristin Fenske: Wiebke.Fenske@bergmannsheil.de.

## Data Availability

The data presented in this study are available upon request from the corresponding author.

## Supplementary Materials

The following supporting information can be downloaded at https://www.mdpi.com/article/10.3390/ijms27020917/s1.

## Abbreviations

2-DG, 2-deoxyglucose; β-AR, beta-adrenergic receptor; ABx, antibiotics; AIMD, antibiotic-induced microbial depletion; AMPK, AMP-activated protein kinase; ATP, adenosine triphosphate; BAT, brown adipose tissue; BAAT, bile acid-CoA:amino acid N-acyltransferase; BSH, bile salt hydrolases; CA, cholic acid; cAMP, cyclic adenosine monophosphate; CDO, cysteine dioxygenase; Cys, cysteine; DIO2, Type II iodothyronine deiodinase; eREE, estimated resting energy expenditure; FMT, fecal microbiota transfer; FCCP, carbonyl cyanide 4-(trifluoromethoxy)-phenylhydrazone; Gαs, G-protein stimulatory α-subunit; GABA_A_, γ-aminobutyric acid receptor; Gly, glycine; HEK, human embryonic kidney; HF, high-fat diet; HF-ABx, antibiotic-treated high-fat-diet-fed rats; iBAT, interscapular brown adipose tissue; KO, knockout; LCA, litocholic acid; Met, methionine; NE, norepinephrine; NEFAs, non-esterified fatty acids; OCR, oxygen consumption rate; PAT1 (SLC36A1), proton-coupled amino acid transporter 1; PCA, principal component analysis; PKA, protein kinase A; PGC1a, peroxisome proliferator-activated receptor gamma coactivator 1-alpha; PPARg, peroxisomal proliferator-activated receptor gamma; PRDM16, PR domain-containing 16 transcription factor; RYGB, Roux-en-Y gastric bypass; SC, standard chow diet; SC-ABx, antibiotic-treated standard-chow-diet-fed rats; SCFAs, short-chain fatty acids; sWAT, subcutaneous white adipose tissue; T4, tetraiodothyronine; T3, triiodothyronine; TauT, taurine transporter (SLC6A6, solute carrier family 6 member 6); TCA, taurocholic acid; TGR5, Takeda G protein-coupled receptor 5; TLCA, taurolithocholic acid; UCP1, uncoupling protein 1; vWAT, visceral white adipose tissue; WT, wild type; WB, Western blot.
