# Supplementary material for: Antibiotic-Mediated Modulation of the Gut Microbiome Identifies Taurine as a Modulator of Adipocyte Function Through TGR5 Signaling
Source: Int J Mol Sci. 2026 Jan 16;27(2):917. doi: 10.3390/ijms27020917 (PMC12842370; doi:10.3390/ijms27020917)
Supplement: Supplementary file 1 [file ijms-27-00917-s001.zip › ijms-3990374-supplementary.pdf]

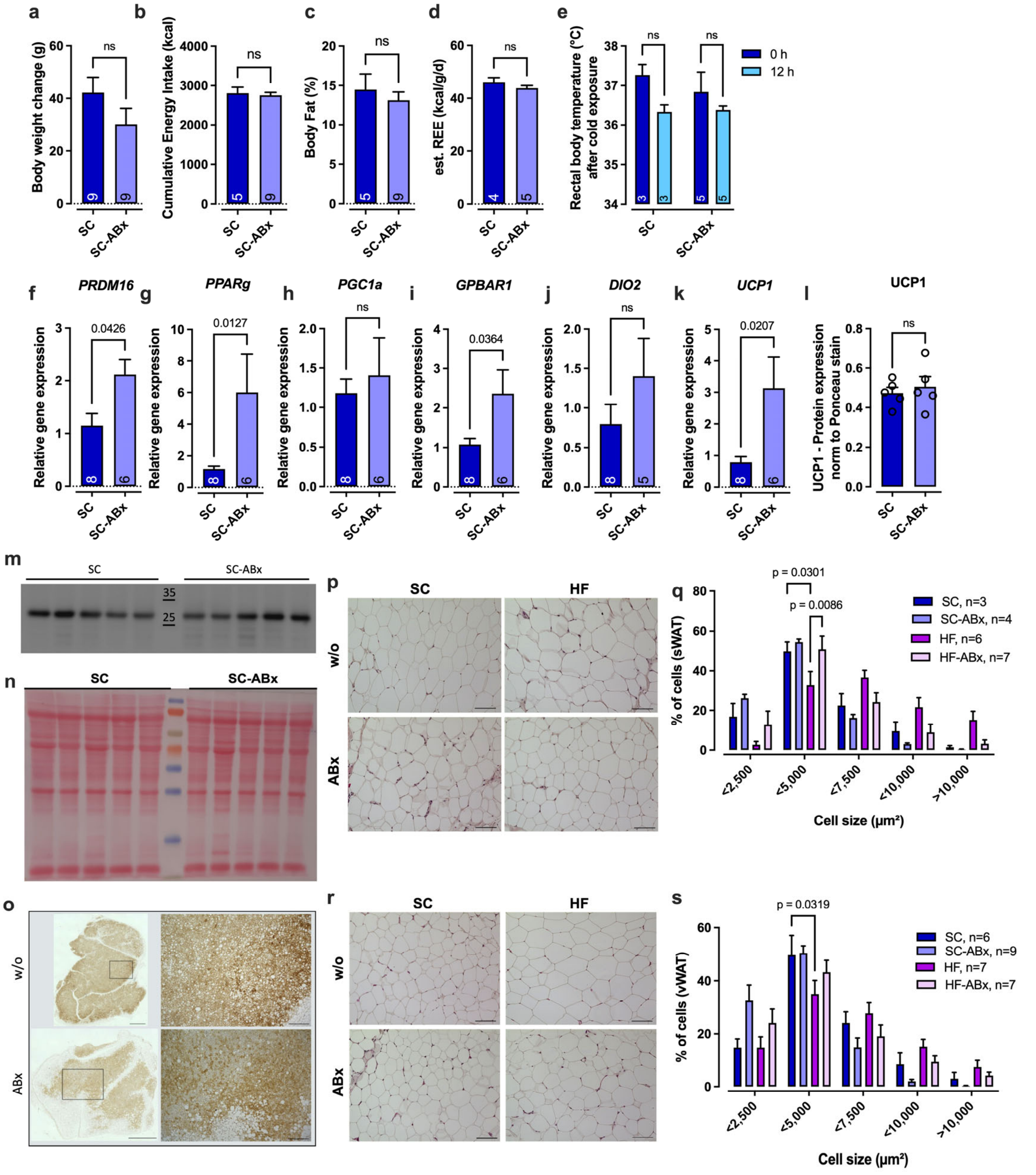

Supplementary Figure S1. (a-o) Male Wistar rats were fed with standard chow diet (SC) for 12 weeks and drinking water was optionally supplemented with an antibiotic cocktail (ABx) for 35d at the end of the experimental setup. Bars indicate Mean+SEM, sample sizes as indicated within bars reflect numbers of tissue from different animals. Mann-Whitney, unpaired t-test (if normal distribution) or ANOVA testing was used to calculate p values as indicated in graphs; (a) Body weight change 35 d after indicated treatment; (b) Cumulative energy intake over 35 d of treatment; (c) Body fat measurement after 35 d of antibiotics treatment; (d) estimated resting energy expenditure (eREE); (e) Rectal body temperature after cold exposure for 12h at 4°C; (f-k) Gene expression analysis via RT-qPCR of thermogenesis marker genes in brown adipose tissue (BAT) using beta-actin as reference gene, relative gene expression indicates comparison of SC-ABx to SC group; (l) Quantification of UCP1 protein expression shown as Western Blot in m; (m) Western Blot to detect UCP1 protein expression (lines and numbers show molecular weight marker in kDa, Ponceau stain used as loading control, n); (o) immunohistochemistry stain for UCP1 in brown adipose tissue; (p-s) H&E immunohistochemistry staining of subcutaneous (sWAT, p,q) and visceral white adipose tissue (vWAT, r,s), shown is one representative image, scale bar 100 μm; (p,r); cell size distribution analysis of sWAT (q) and vWAT (s).

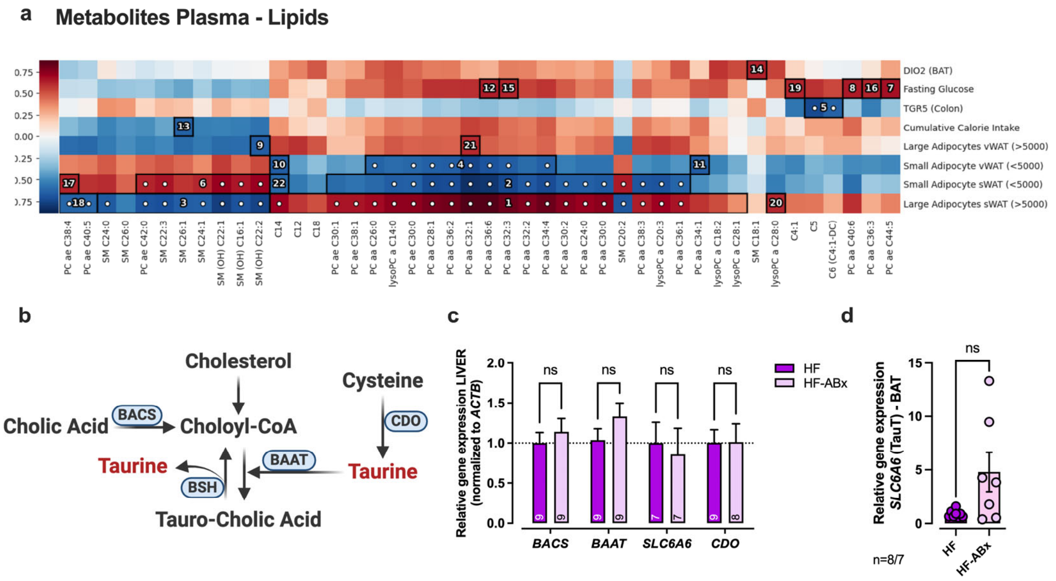

Supplementary Figure S2. (a-d) Male Wistar rats were fed with high-fat diet (HF) and drinking water was optionally supplemented with an antibiotics cocktail (ABx). (a) Metabolomics analysis was performed from plasma samples, Spearman's rank correlation study of phenotypic features and lipids in plasma, top 30 correlations are indicated with numbers as rankings, white dots are significant correlations, red tiles are positive correlations, blue tiles are negative correlations, tiles without the white dots are trends but don't reach significance, frames indicate equal rank correlation; (b) schematic simplified overview of taurine metabolism and involved enzymes (in boxes); Created in BioRender. Jaeger, E. (2025) https://BioRender.com/6wehdhy; (c) analysis of gene expression in liver of enzymes involved in taurine metabolism and transport BACS, BAAT, TauT, CDO by RT-qPCR; (d) gene expression analysis of the taurine transporter TauT in brown adipose tissue (BAT) via RT-qPCR using beta-Actin as reference gene. If not stated otherwise Mann-Whitney or ANOVA testing was used to calculate p-values. BACS - ATP-dependent microsomal BA coenzyme A (CoA) synthetase; BAAT - BA-CoA:amino acid N-acetyltransferase; BSH – bile salt hydrolase, CDO – cysteine dioxygenase.

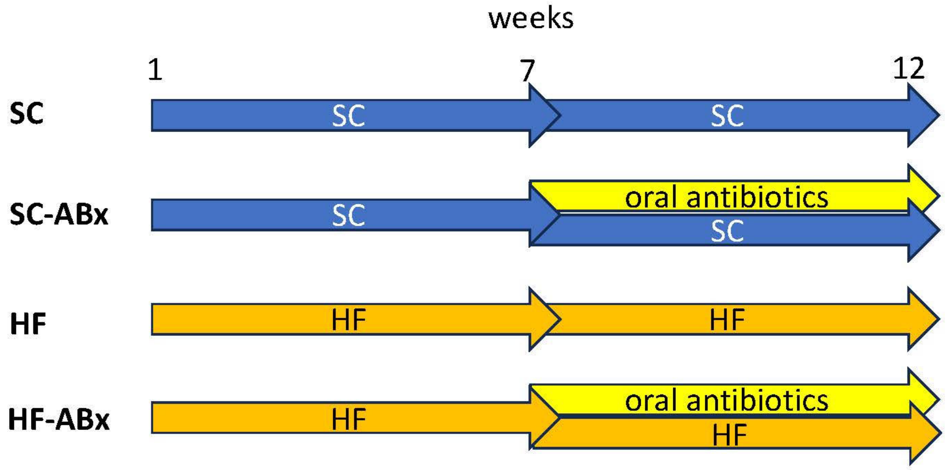

Supplementary Figure S3. Overview of experimental design. SC – standard chow, HF – high fat diet, ABx – antibiotic treatment.
